# Neuropharmacology of New Psychoactive Substances (NPS): Focus on the Rewarding and Reinforcing Properties of Cannabimimetics and Amphetamine-Like Stimulants

**DOI:** 10.3389/fnins.2016.00153

**Published:** 2016-04-19

**Authors:** Cristina Miliano, Giovanni Serpelloni, Claudia Rimondo, Maddalena Mereu, Matteo Marti, Maria Antonietta De Luca

**Affiliations:** ^1^Department of Biomedical Sciences, University of CagliariCagliari, Italy; ^2^Advisory and Steering Group, URITo.N. - Unit for Research and Innovation on Forensic Toxicology, Neuroscience of Addiction and New Drugs. FT-DSS University of FlorenceFlorence, Italy; ^3^Department of Diagnostic and Public Health, University of VeronaVerona, Italy; ^4^Departmentof Pharmaceutical and Pharmacological Sciences, University of PaduaPadua, Italy; ^5^Department of Life Sciences and Biotechnology, University of FerraraFerrara, Italy

**Keywords:** novel psychoactive substances, NPS, cannabinoids, psychostimulants, JWH-018, Spice

## Abstract

New psychoactive substances (NPS) are a heterogeneous and rapidly evolving class of molecules available on the global illicit drug market (e.g smart shops, internet, “dark net”) as a substitute for controlled substances. The use of NPS, mainly consumed along with other drugs of abuse and/or alcohol, has resulted in a significantly growing number of mortality and emergency admissions for overdoses, as reported by several poison centers from all over the world. The fact that the number of NPS have more than doubled over the last 10 years, is a critical challenge to governments, the scientific community, and civil society [EMCDDA (European Drug Report), 2014; UNODC, 2014b; Trends and developments]. The chemical structure (phenethylamines, piperazines, cathinones, tryptamines, synthetic cannabinoids) of NPS and their pharmacological and clinical effects (hallucinogenic, anesthetic, dissociative, depressant) help classify them into different categories. In the recent past, 50% of newly identified NPS have been classified as synthetic cannabinoids followed by new phenethylamines (17%) (UNODC, 2014b). Besides peripheral toxicological effects, many NPS seem to have addictive properties. Behavioral, neurochemical, and electrophysiological evidence can help in detecting them. This manuscript will review existing literature about the addictive and rewarding properties of the most popular NPS classes: cannabimimetics (JWH, HU, CP series) and amphetamine-like stimulants (amphetamine, methamphetamine, methcathinone, and MDMA analogs). Moreover, the review will include recent data from our lab which links JWH-018, a CB1 and CB2 agonist more potent than Δ^9^-THC, to other cannabinoids with known abuse potential, and to other classes of abused drugs that increase dopamine signaling in the Nucleus Accumbens (NAc) shell. Thus the neurochemical mechanisms that produce the rewarding properties of JWH-018, which most likely contributes to the greater incidence of dependence associated with “Spice” use, will be described (De Luca et al., 2015a). Considering the growing evidence of a widespread use of NPS, this review will be useful to understand the new trends in the field of drug reward and drug addiction by revealing the rewarding properties of NPS, and will be helpful to gather reliable data regarding the abuse potential of these compounds.

## Introduction

**Figure 1:**
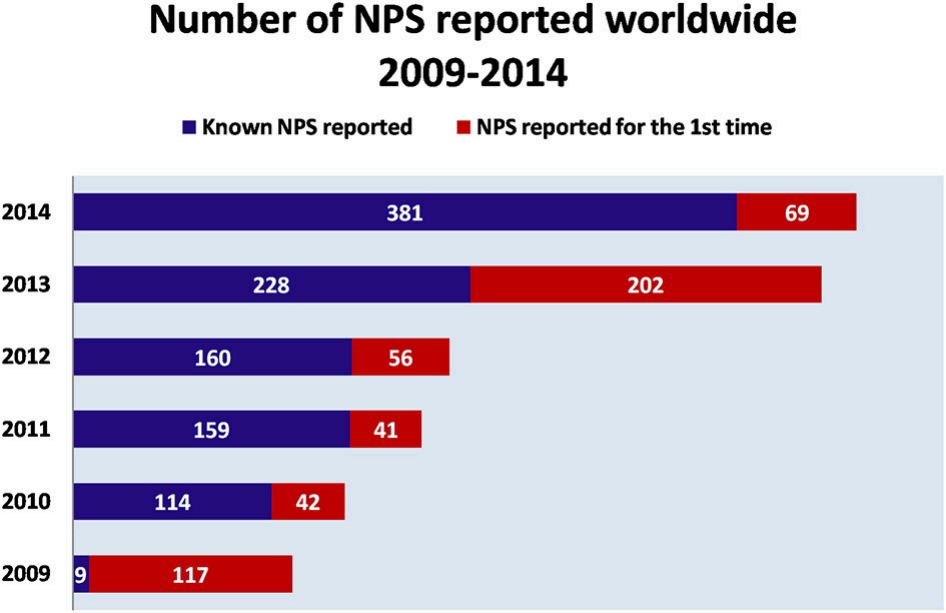
**Number of NPS reported worldwide (2009–2014)**. Adapted from UNODC (2014a). 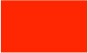 NPS reported for the first time 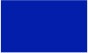 Known NPS reported.

Over the last decade, New Psychoactive Substances (NPS) have become a global phenomenon. The emergence of these substances have been reported in almost 100 countries and territories, and more than 500 NPS have been identified worldwide based on reports by national governments, as well as the EU, and international institutions (UNODC, 2014a, 2015) (Figure 1). In 2014, in Europe alone, 101 NPS have been detected showing an increase of 25%, as compared to 2013 [EMCDDA (New psychoactive substances in Europe), 2015b]. NPS are able to mimic the effects of controlled substances and are mainly synthetic cannabinoids, stimulants, hallucinogens, and opioids.

Previous studies show that the use of NPS occurs among different subject groups: school students, partygoers, psychonauts, prisoners, and injecting drug users. Motivations for use include factors such as legal status, availability, and cost, as well as the desire to avoid detection and user preferences for particular pharmacological properties [González et al., 2013; Helander et al., 2013, 2014; EMCDDA (European Drug Report), 2015a; EMCDDA (New psychoactive substances in Europe), 2015b]. Although global research is not available yet about NPS, prevalence of use among the population, single national surveys (with respect to substances and subpopulations) show that the use of NPS amongst the general adult population is relatively low compared with the use of other illicit drugs. However, adolescents use more NPS than illicit drugs mostly because many of them are legal and easily available on the web [Drug Policy Department Italian Presidency of the Council of Ministers, 2014; Fraser, 2014; Kikura-Hanajiri et al., 2014; EMCDDA (New psychoactive substances in Europe), 2015b; Hondebrink et al., 2015; Palamar et al., 2015; Wood et al., 2015].

Similar to many known illicit drugs, NPS can cause severe physical and psychological symptoms that can even result in death [Drug Policy Department Italian Presidency of the Council of Ministers, 2013b; Fraser, 2014; EMCDDA (European Drug Report), 2015a; UNODC, 2015]. A recent analysis by the European Drug Emergencies Network, monitoring emergency admissions in the last 5 years in 10 EU countries, found that 9% of all drug-related emergencies involved NPS, primarily synthetic cannabinoids and cathinones (Wood et al., 2014). Consequently, there is increasing evidence that NPS play a great role in hospital emergencies and some drug-induced deaths [EMCDDA (European Drug Report), 2015a]. However, the analytical detection of NPS for emergency services is not technically available so far; their recognition by means of second level analysis requires standards solution, methodologies and analytical equipment not accessible to every laboratory yet [Drug Policy Department Italian Presidency of the Council of Ministers, 2013b; UNODC, 2014a; EMCDDA (European Drug Report), 2015a]. Therefore, their identification in biological samples, as well as in seized or collected samples, represents one major difficulty.

Notably, the internet is an important marketplace for the sale of NPS. Evidence is emerging of so-called “gray marketplaces”-online sites selling NPS which operate on both the surface as well as the deep web (Deluca et al., 2012; Drug Policy Department Italian Presidency of the Council of Ministers, 2013b; Burns et al., 2014; Corazza et al., 2014). Therefore, NPS can be sold via the internet to everyone, including young, underage teenagers, with complete anonymity and an easy avoidance of law enforcement and health controls [Drug Policy Department Italian Presidency of the Council of Ministers, 2013b; UNODC, 2014a; EMCDDA (European Drug Report), 2015a]. The growth of online and virtual drug markets strongly contributes to the uncontrolled widespread use of these substances, increasing health risks for consumers, and challenging drug control policies.

The largest increase in terms of newly reported NPS involves synthetic cannabinoids, synthetic cathinones, and phenethylamines [EMCDDA (European Drug Report), 2015a; EMCDDA (New psychoactive substances in Europe), 2015b]. The first synthetic cannabinoids were identified in 2008 in preparations called “herbal mixtures” or “herbal blends” (i.e., Spice) and sold as incense or air fresheners. Their effects are similar, if not superior, to those caused by cannabis consumption (Hohmann et al., 2014; Khullar et al., 2014; Mills et al., 2015). Recently, a survey of the use of synthetic cannabinoids among US students showed that Spice products were the second most used drug after marijuana, with a prevalence of 7.4–7.9% in those aged between 15 and 18 years (Johnston et al., 2013). Adolescence, a critical developmental period commonly associated with an increase in drug abuse in the human population, may be a stage of particular vulnerability to the effects of the new psychoactive drugs (Johnston et al., 2013). In fact, most of the brain receptor systems have been shown to mature slowly, reaching maximal levels around age 20. Indeed, the use of these drugs might influence neurodevelopment inducing psychiatric disorders or other mental deficits (Paus, 2005; Sussman et al., 2008). Several NPS such as mephedrone, pentedrone and MDPV which mimic the effects of amphetamine-like stimulants (ATS), can be classified as synthetic cathinones with stimulant and empathogenic properties, or as phenethylamines which can induce stimulant and hallucinogenic effects (UNODC, 2014a, 2015). Similar to other NPS, synthetic cannabinoids and ATS are largely available online and are often sold as research chemical components. They are mainly produced in Eastern Europe, Central Asia and China, and then shipped and sold to Europe and the USA (UNODC, 2015).

Currently, not all NPS are under international control. Many countries worldwide have established permanent control measures for some substances or issued temporary bans [EMCDDA (New psychoactive substances in Europe), 2015b; UNODC, 2015]. Only a few NPS have been reviewed by the mechanisms established under the international drug conventions. Existing laws covering issues unrelated to controlled drugs, such as consumer safety legislation, have been used in some countries such as Poland and UK; in others (Hungary, Finland, Italy, France, Denmark, etc.) existing drug laws or processes have been extended or adapted; additionally, in Ireland, Austria, Portugal, Romania, and Sweden new legislation has been designed [EMCDDA (New psychoactive substances in Europe), 2015b; UNODC, 2015].

The forensic identification of NPS is very difficult. These may concern the lack of knowledge on NPS available to the professionals performing analytical analysis. In addition, analytical methodologies are still not sufficient to detect the presence of all of the NPS in the analyzed samples and many laboratories lack appropriate analytical equipment for their recognition (Drug Policy Department Italian Presidency of the Council of Ministers, 2013a). These are all important aspects to take into account when considering the legal, health, and social consequences related to NPS.

To date, several behavioral, neurochemical, and electrophysiological studies have helped us to understand the pharmacological mechanisms of action of NPS. However, many of them have been focused on the acute toxicological consequences of NPS use. As they are relatively new and novel, there are no epidemiological studies to show the long-term effects of these psychoactive compounds. Also, there is not a lot of evidence on the addictive properties of NPS.

This work has been divided into two main parts based on pharmacological classification of the most popular and public health-concerning NPS classes: amphetamine-like stimulants and cannabimimetic drugs. Moreover, specific references to recent papers by the authors have been presented. A thorough analysis of the rewarding and reinforcing properties of NPS and their abuse liability will hopefully, provide to be useful for understanding the new disturbing trends in the field of drug addiction and provide strategies to tackle this growing problem.

## NPS: from chemistry to pharmacological effects

NPS can be divided into six chemical classes (Martinotti et al., 2015; Schifano et al., 2015): *phenethylamines, piperazines, tryptamines, synthetic cathinones, alkylindoles (synthetic cannabinoids) and arylcyclohexylamines* (see Table 1). Alternatively, a different classification is based on pharmacological and clinical effects: stimulants, entactogens, hallucinogens, and cannabis-like compounds.

**Table 1:** **New Psychoactive Substances (NPS) classification**.

**Chemical class**	**Pharmacological effects**	**References**
*Phenethylamines*	Serotoninergic receptor agonists that cause psychedelic effects and inhibit monoamine reuptake	Nelson et al., 2014
	Effects: Hypertension, vomiting, hyperthermia, convulsions, dissociation, hallucinations, respiratory deficits, liver and kidney failure, and death in case of overdose	Winstock and Schifano, 2009; Schifano et al., 2010; Corazza et al., 2011; Bersani et al., 2014
*Piperazines*	Stimulants that promote the release of dopamine and noradrenaline and inhibits the uptake of monoamines	Kersten and McLaughlin, 2015; Smith et al., 2015
	Effects: Hyperthermia, convulsions, and kidney failure; hallucinations and death have been reported at high doses	
*Tryptamines*	5HT2A receptor agonists and serotonin reuptake inhibitors	Lessin et al., 1965; Nichols, 2004; Sogawa et al., 2007; Fantegrossi et al., 2008; Cozzi et al., 2009; Fontanilla et al., 2010
	Effects: Visual hallucinations, alterations in sensory perception, depersonalization	
*Synthetic cathinones*	Sympathomimetic drugs that act on serotonin, dopamine, and noradreline pathways	Corkery et al., 2012, 2014; Schifano et al., 2012; Loi et al., 2015
	Effects: Agitation, restlessness, vertigo, abdominal pain, paranoia, rhabdomyolysis, convulsions, and death	
*Synthetic cannabinoids*	CB1 and CB2 receptors agonists displaying higher affinity, efficacy and potency compared to Δ^9^-THC	Fattore and Fratta, 2011; Brents and Prather, 2014; De Luca et al., 2015a,b
	Effects: Euphoria, anxiolytic, and antidepressant-like effects, paranoia, tachycardia, panic, convulsions, psychosis, visual/auditory hallucinations, vomiting, and seizures	Hermanns-Clausen et al., 2013; Winstock and Barratt, 2013
*Arylcyclohexylamine*	Dissociative anesthetics that act as 5HT2A agonist and NMDA receptor antagonist and show high affinity for opioid receptors	Nishimura and Sato, 1999; ACMD (Advisory Council on the Misuse of Drugs), 2013; Schifano et al., 2015
	Effects: Distort perceptions of sight and sound, dissociation from the environment and selfwithout hallucinations	

*Phenethylamines, piperazines, tryptamines, and synthetic catinones* exhibit stimulant and hallucinogenic effects, making up the distinct class of entactogens, which are described as psychoactive substances that enhance feelings of empathy, love, and emotional closeness to others (Schifano et al., 2007). Entactogens can be chemically divided into phenethylamines, amphetamines, synthetic cathinones, piperazines, pipradrols/piperidines, aminoindanes, benzofurans, and tryptamines (see Table 2). Stimulant drugs usually inhibit monoamine reuptake, increasing the quantity of noradrenaline, dopamine and serotonin in the synaptic cleft leading to sympathomimetic effects (Schifano, 2013). *Phenethylamines* are synthetic compounds commercially known as “party pills” (e.g., tablets of different colors/shapes, capsules, powder/crystal). They act on serotoninergic receptors leading to psychedelic effects and some of them inhibit the monoamine reuptake as well (Nelson et al., 2014); 3,4-methylenedioxy-methamphetamine (MDMA), widely known as “ecstasy,” is one of the most popular drugs among young people because of its stimulant effects. But, recently a growing use of new dangerous molecules on the recreational drug scene, such as 2C and its derivatives (e.g., “N-Bomb,” “B-Fly,” and “Dr. Death”), 2-D series drugs, 3C-bromo-Dragonfly, 4-MTA, 6-APB, 4,4′-DMAR and MPA, that are novel derivatives of classic psychedelic phenethylamines/MDMA-like drugs (Nelson et al., 2014) has been reported; several cases of intoxications have been reported with symptoms such as hypertension, vomiting, hyperthermia, convulsions, dissociation, hallucinations, respiratory deficits, liver, and kidney failure and death in case of overdose (Winstock and Schifano, 2009; Schifano et al., 2010; Corazza et al., 2011; Dean et al., 2013; Bersani et al., 2014; Le Roux et al., 2015; Maas et al., 2015). The lead compound in *piperazines*, N-Benzylpiperazin (BZP), has a typical central nervous system stimulant structure so it triggers the release of dopamine and norepinephrine and inhibits the uptake of dopamine, norepinephrine and serotonin (Smith et al., 2015). Although BZP is structurally similar to amphetamine, it is reported to have only one-tenth the potency (Wikström et al., 2004). However, at higher dosages, hallucinations can be reported as well (Kersten and McLaughlin, 2015). Before legal restrictions were placed on it, BZP was used as a safe alternative to amphetamines such as MDMA (Monteiro et al., 2013). *Tryptamines* (the most common is the lysergic acid diethylamide-LSD) are a group of monoamine alkaloids, very similar to the endogenous neurotransmitter serotonin (5-hydroxytryptamine, 5-HT) (Tittarelli et al., 2015), so they act both as 5HT2A receptor agonists and serotonin reuptake inhibitors (Lessin et al., 1965; Nichols, 2004; Fantegrossi et al., 2008; Cozzi et al., 2009; Fontanilla et al., 2010) provoking visual hallucinations, alterations in sensory perception, and depersonalization (Sogawa et al., 2007); novel tryptamines, as 5-MeO-AMT or 5-MeO-DMT, continue to appear on the online drug market and on the “dark net” (Araújo et al., 2015; Schifano et al., 2015; Teixeira-Gomes et al., 2014). *Synthetic cathinones* (mephedrone, methylone,butylone, MDPV, and α-PVP) are structural analogs of cathinones (a molecule present in the psychoactive plant Khat) and are available in tablets, capsules, powder/crystal and generally labeled as “bath salts” or “plant fertilizers” (Fass et al., 2012; German et al., 2014; Valente et al., 2014; Karila et al., 2015). Clinical effects most commonly reported with cathinones include anxiety, impaired concentration and memory, irritation of the nasal mucosa, headache, tachycardia, and hypertension. The typical clinical symptoms are indistinguishable from the acute effects of MDMA or cocaine (Prosser and Nelson, 2012; Baumann et al., 2013; Valente et al., 2014); among their psychoactive effects, agitation, restlessness, vertigo, abdominal pain, paranoia, rhabdomyolysis, convulsions, and death are included (Corkery et al., 2012, 2014; Schifano et al., 2012; Loi et al., 2015).

**Table 2:** **Chemical classes of stimulant drugs**.

**Chemical group**	**Representatives**		
	**Usual name**	**Chemical name**	**References**
Phenethylamines	2-PEA	2-phenylethanamine	Teixeira-Gomes et al., 2014
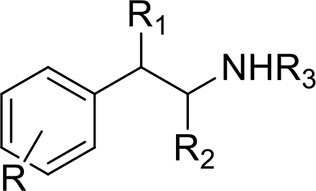	DMMA	2-(3,4-dimethoxyphenyl)-Nmethylpropan-2-amine	
	DMA	N,N-dimethyl-1-phenylpropan-2-amine	UNODC, 2013
	β-Me-PEA	2-phenylpropan-1-amine	
	Phenpromethamine	N-methyl-2-phenylpropan-1-amine	Liechti, 2015
			Schifano et al., 2015
Amphetamines	PMMA	1-(4-methoxyphenyl)-N-methylpropan-2-amine	Iversen et al., 2014
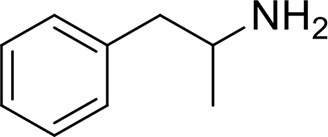	PMA	1-(4-methoxyphenyl)propan-2-amine	
	4-FMA	1-(4-fluorophenyl)-N-methylpropan-2-amine	Zawilska, 2015
	4-CA	1-(4-chlorophenyl)propan-2-amine	
	2-FA	1-(2-fluorophenyl)propan-2-amine	Simmler et al., 2014
	2-FMA	1-(2-fluorophenyl)-N-methylpropan-2-amine	
	Fenfluramine	3-trifluoromethyl-N-ethylamphetamine	
Synthetic cathinones or beta-keto (bk) amphetamines	4-MMC	(RS)-1-(4-methylphenyl)-2-methylaminopropan-1-one	Baumann et al., 2013
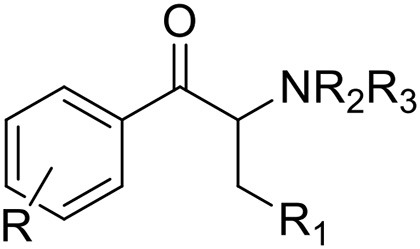	4-EMC	1-(4-ethylphenyl)-2-(methylamino)propan-1-one	
	3,4-DMMC	1-(3,4-dimethylphenyl)-2-(methylamino)propan-1-one	Kelly, 2011
	Pentedrone	2-(methylamino)-1-phenylpentan-1-one	
	Mephedrone	2-(methylamino)-1-phenylpentan-1-one	Coppola and Mondola, 2012
	Metilone	1-(1,3-benzodioxol-5-yl)-2-(methylamino)propan-1-one	
	MDPV	1-(1,3-benzodioxol-5-yl)-2-pyrrolidin-1-ylpentan-1-one	Paillet-Loilier et al., 2014
	αPVP	1-phenyl-2-pyrrolidin-1-ylpentan-1-one	
	bk-PMMA	1-(4-methoxyphenyl)-2-(methylamino)propan-1-one	Schifano et al., 2015
			Simmler et al., 2013
Piperazines	BZP	N-benzylpiperazine	Iversen et al., 2014
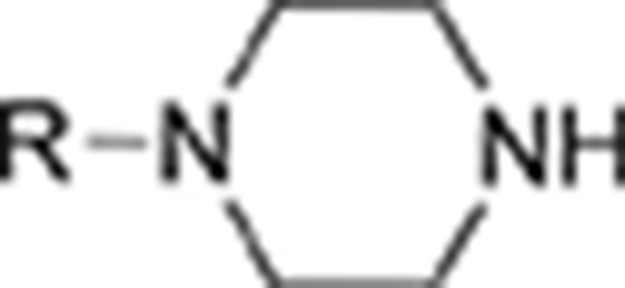	pCPP	1-(4-chlorophenyl)-piperazine	
	mCPP	1-(3-chlorophenyl)-piperazine	Zawilska, 2015
	2C-B-BZP	1-[(4-bromo-2,5-dimethoxyphenyl)methyl]piperazine	
	TFMPP	1-(3-trifluoromethylphenyl)-piperazine	UNODC, 2013
	MeOPP	4-methoxyphenylpiperazine	
	pFPP	4-fluorophenylpiperazine	
Pipradrols/Piperidines	2-DPMP	2-(Diphenylmethyl)piperidine	Zawilska, 2015
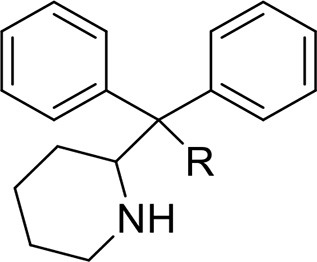	desoxy-D2PM	2-(Diphenylmethyl)pirrolidine	
			Liechti, 2015
			UNODC, 2013
Aminoidanes	2-AI	2,3-dihydro-1H-inden-2-amine	Iversen et al., 2014
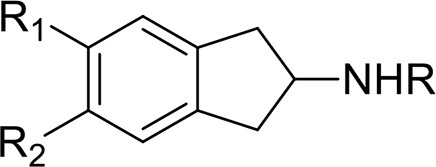	5-IAI	5-iodo-2,3-dihydro-1H-inden-2-amine	
	MDAI	6,7-Dihydro-5H-cyclopenta[f][1,3]benzodioxol-6-amine	UNODC, 2013
	MMDAI	5,6-Methylenedioxy-N-methyl-2-aminoindane	
	MDAT	6,7- Methylenedioxy-2-aminotetralin	
Benzofurans	5-APB	5-(2-aminopropyl)benzofuran	Iversen et al., 2013
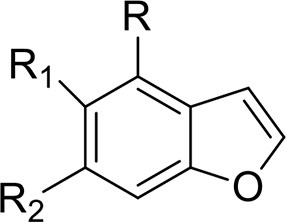	5-APDB	1-(2,3-dihydro-1-benzofuran-5-yl)propan-2-amine	
	5-MAPB	1-(benzofuran-5-yl)-N-methylpropan-2-amine	Iversen et al., 2014
	6-APB	6-(2-aminopropyl)benzofuran	
	6-APDB	1-(2,3-dihydro-1-benzofuran-6-yl)propan-2-amine	Corkery et al., 2013
Tryptamines	AMT	1-(1H-indol-3-yl)propan-2-amine	Schifano et al., 2015
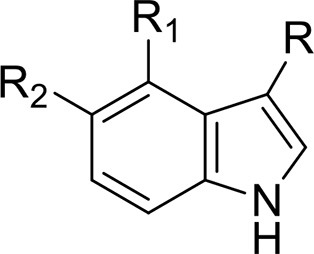	5-IT, 5-API	1-(1H-indol-5-yl)propan-2-amine	
	5-APDI	1-(2,3-Dihydro-1H-inden-5-yl)-2-propanamine	Teixeira-Gomes et al., 2014
	4-AcO-DPT	4-Acetoxy-N,N-dipropyltryptamine	
	5-MeO-DPT	5-methoxy-N,N-dipropyltryptamine	Araújo et al., 2015
	4-AcO-DMT	4-acetoxy-N,N-dimethyltryptamine	
	4-AcO-DALT	4-Acetoxy-N,N-diallyltryptamine	
	5-MeO-AMT	5-methoxy-α-methyltryptamine	
	5-MeO-DMT	5-metossi-N,N-dimetiltriptamina	
2C Agents-substituted phenylethylamines	2C-H	2,5-dimethoxyphenethylamine	Eshleman et al., 2014
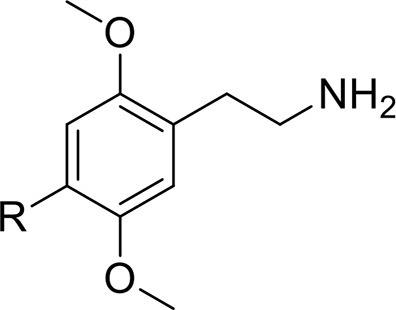	2C-B	4-bromo-2,5-dimethoxyphenethylamine	
	2C-E	2,5-dimethoxy-4-ethylphenethylamine	Schifano et al., 2015
	2C-N	2,5-Dimethoxy-4-nitrophenethylamine	
	2C-G	2-(2,5-dimethoxy-3,4-dimethylphenyl)ethanamine	Welter-Luedeke and Maurer, 2015
2D Agents-substituted phenylethylamines	DOI	1-(4-iodo-2,5-dimethoxyphenyl)-propan-2-amine	Zawilska, 2015
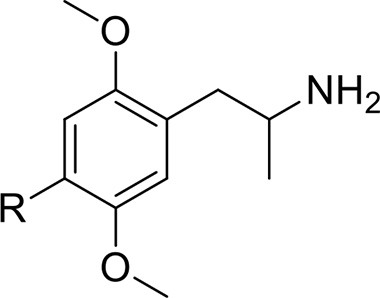	DOC	1-(4-chloro-2,5-dimethoxyphenyl)-propan-2-amine	
	DOB	1-(4-bromo-2,5-dimethoxyphenyl)propan-2-amine	Gatch et al., 2009
	DOM	2,5-Dimethoxy-4-methylamphetamine	
NBome Agents-substituted phenylethylamines	25H-NBOMe	1-(2,5-dimethoxyphenyl)-*N*-[(2-methoxyphenyl)methyl]ethanamine	Zawilska, 2015
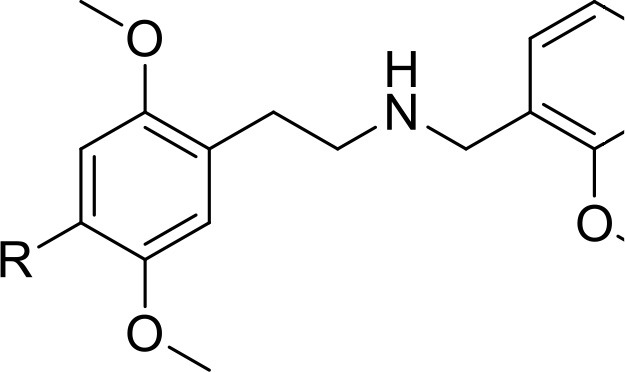	25I-NBOMe	4-iodo-2,5-dimethoxy-N-(2-methoxybenzyl)phenethylamine	
	25B-NBOMe	2-(4-bromo-2,5-dimethoxyphenyl)-N-[(2-methoxyphenyl)methyl]ethanamine	Schifano et al., 2015
	25E-NBOMe	2-(2,5-dimethoxy-4-ethylphenyl)-N-(2-methoxybenzyl)ethanamine	
	25N-NBOMe	2-(2,5-Dimethoxy-4-nitrophenyl)-N-(2-methoxybenzyl)ethanamine	Kyriakou et al., 2015

**Figure 2:**
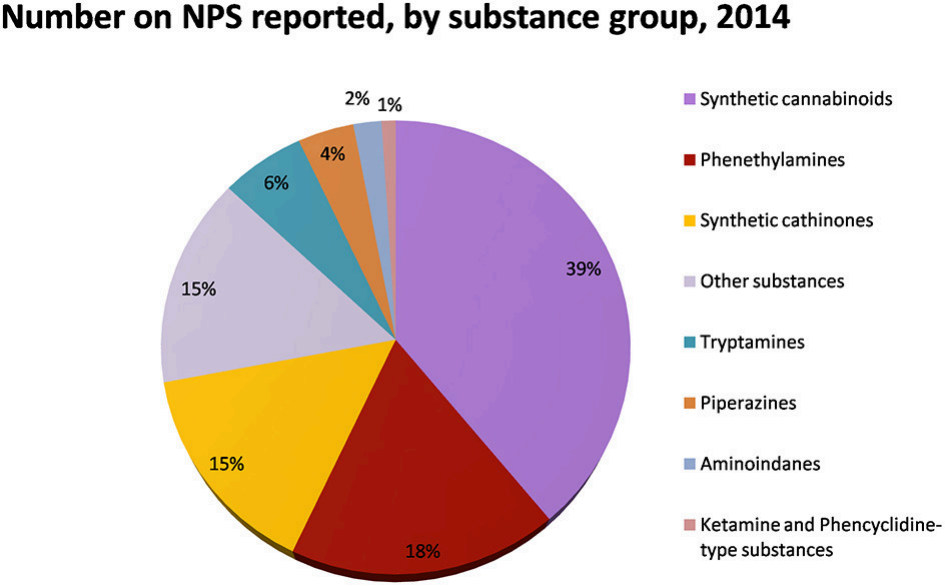
**Number of NPS reported by substance group in 2014**. Adapted from UNODC (2014a).

Synthetic cannabinoids belong to the *alkylindoles* and *cyclohexylphenos* classes which show high affinity for CB1 and CB2 cannabinoid receptors and act like Δ^9^-THC but with prolonged psychoactive effects and more side effects (Fattore and Fratta, 2011; Brents and Prather, 2014). As shown in Figure 2, they can be divided into naphtoylindoles (e.g., JWH- 018, JWH-073, JWH-210, WIN-55212), phenylacetylindoles (e.g., JWH-250 e JWH-251), benzoylindoles (e.g., WIN-48,098, AM-694, RSC-4), cyclohexylphenols (e.g., CP-47497, CP-55940, CP-55244) (Smith et al., 2015). They are generally consumed by inhalation through the consumption of cigarettes containing herbal substances as well as these synthetic molecules to obtain euphoria, anxiolytic, and antidepressant-like effects. However, reports presented by the EMCDDA (2009a) and by the Italian Early Warning System – N.E.W.S. (Anti-drug Policies Department) have shown effects like paranoia, tachycardia, panic, convulsions, psychosis, visual/auditory hallucinations, vomiting, and seizures (Hermanns-Clausen et al., 2013; Winstock and Barratt, 2013).

Finally, *arylcyclohexylamine* (ketamine, phencyclidine- PCP and methoxetamine) are dissociative anesthetics that distort perceptions of sight and sound and produce feelings of detachment (or dissociation) from the environment and self without hallucinations [Nishimura and Sato, 1999; ACMD (Advisory Council on the Misuse of Drugs), 2013]. Although present in the classification, the rewarding properties of the latter group will not be discussed in this review paper.

## Human and animal studies on amphetamine-like stimulants effects: psychoactive effects, cognitive deficits, emotional alterations, and dependence

In the second part of the 90s, a global trend of escalating amphetamine-like stimulant use was observed and synthetic tryptamines appeared on illicit drug markets. Instead of replacing or displacing MDMA and cocaine, mephedrone, and other NPS from this group appear to have been added to the established repertoire of psychostimulant narcotics (Sanders et al., 2008; Zawilska, 2015).

In animal models of addiction, cathinones have displayed potential rewarding and reinforcing effects. For example, mephedrone produces conditioned place preference (CPP), facilitates intracranial self-stimulation and is self-administered in rats (Hadlock et al., 2011; Lisek et al., 2012; Motbey et al., 2013; Bonano et al., 2014; Gregg et al., 2015). Prior studies demonstrated that MDPV and methylone, another synthetic cathinone, increase locomotor activity in rodents (López-Arnau et al., 2012; Marusich et al., 2012; Aarde et al., 2013; Gatch et al., 2013) and also enhance intracranial self-stimulation (Watterson et al., 2012, 2014; Bonano et al., 2014) and engender conditioned place preference (Karlsson et al., 2014), effects that are indicators of high abuse potential (Schindler et al., 2015). This evidence suggests that each compound could produce behavioral effects consistent with psychostimulant drugs displaying high abuse liability, possibly higher than amphetamine. In fact, in rats trained to self-administer MDPV or methamphetamine, dose-substitution studies demonstrated that behavior was dose-sensitive for both drugs, but MDPV showed greater potency and efficacy than methamphetamine (Paillet-Loilier et al., 2014). Moreover, in mice models, mephedrone, methylone, and MDPV produce CPP equal or higher than amphetamine, strongly suggesting their addictive properties (Karlsson et al., 2014). In addition, MDMA, methylone, and mephedrone are self-administered in female rats with a higher intake in mephedrone-trained rats compared to methylone-trained animals. This seems to suggest that mephedrone might have greater reinforcing effects compared to methylone or MDMA (Creehan et al., 2015), despite their shared mechanism of action. They are non-selective transporter substrates that increase the release of dopamine, norepinephrine and 5-HT *in vitro* (Baumann et al., 2012; Eshleman et al., 2013; Simmler et al., 2013). Importantly, the 5-HT-releasing ability of these drugs is more prevalent than their effects on dopamine *in vivo* (Baumann et al., 2008, 2012; Kehr et al., 2011; Wright et al., 2012) suggesting empathogen-like effects. Such findings indicate that self-administration of MDMA-like drugs is influenced by 5-HT release, but also drug pharmacokinetics, effects on noradrenergic systems, or non-transporter sites of action (Schindler et al., 2015).

In humans, synthetic cathinones produce psychotropic effects similar to MDMA and cocaine (Simmler et al., 2013). The typical dose range varies according to the different cathinone derivatives. However, according to information released from users in drug forums, where people discuss their experiences with recreational drugs (e.g., “Drugs-Forum,” “Urban 75,” “Erowid”), they usually start with a small dose and gradually increase it. This is in line with scientific reports which show that an excessive increase of noradrenergic signals could promote the onset of adverse effects and that the potency of a substance to activate the noradrenergic system is inversely correlated to the doses typically used recreationally (Simmler et al., 2013). All cathinones exhibit higher dopaminergic activity when compared with their non β-keto amphetamines analogs. Recent studies of the mechanisms by which b-ketoamphetamines interact with DAT, offer significant insight into why these drugs have such divergent effects on neurotoxicity. “Bath salts” have been classified as substrates and non-substrates based on whether or not they are transported by the DAT (Anneken et al., 2015). This increased dopaminergic property of the cathinones suggests higher stimulant-type effects and a greater risk for dependence (Aarde et al., 2013). Stimulant and entactogenic properties are typical of cathinones. In fact, desired or pleasant effects most often described by users include euphoria, intensification of sensory senses, increased sociability, increased energy, mental stimulation, empathy connection, openness, increased sensory perception, decreased inhibition, and sexual arousal; but side effects such as cognitive confusion, cognitive impairment, psychiatric irritability, aggression that sometimes progresses to violent or even criminal behavior, and self-destructive behavior have also been reported [IACP (International Association of Chiefs of Police), 2012]. MDPV and mephedrone have been directly implicated in a number of fatalities in medical literature. In one case involving MDPV, the cause of death was consistent with excited delirium syndrome, a condition associated with stimulant drug overdose and attributable to excessive dopaminergic transmission (Baumann et al., 2012).

However, amphetamine remains the prototype of psychostimulants causing agitation, insomnia, loss of appetite and, at higher doses, “amphetamines psychosis” characterized by paranoia, hallucinations and delusion (Iversen et al., 2014). In experimental animals, low doses of amphetamine cause hyperactivity and higher doses lead to stereotyped repetitive behaviors (Whelpton, 2007). The neurotoxic effects of amphetamines has been vastly studied and their ability to damage brain monoaminergic cells was shown by long-term deficits in dopaminergic and serotoninergic systems in several brain areas of animals (Teixeira-Gomes et al., 2014). One of the major neurotoxic actions of amphetamines observed in laboratory animals is the sustained depletion of monoamine brain levels. In addition to the damage to dopaminergic and serotoninergic neuronal systems, amphetamines can also induce neuronal death. For example, in several studies, MDMA administration in mice and rats produced neuronal death in several brain areas including the cortex, hippocampus, amygdala, ventromedial/ventrolateral thalamus, and teniatecta (Teixeira-Gomes et al., 2014). MDMA is still one of the most widely used recreational drugs and many NPS were designed to mimic its effects or as substitutes for MDMA in ecstasy pills.

The potency of abused psychostimulants to activate the brain reward circuitry increases the risk of potential for abuse and addiction in humans (Table 3). In contrast, a relative activation of the serotonin system would be linked to a reduction in abuse potential. Thus, the DAT/SERT inhibition ratio and dopamine/serotonin release potency has been proposed to predict the effects of psychostimulants in humans (Paillet-Loilier et al., 2014). Data currently available has shown that the frequent consumption of high doses of synthetic cathinones induce tolerance, dependence, craving, and withdrawal syndrome after sudden suspension [NDIC (National Drug Intelligence Center), 2011]. Indeed, Kehr et al. (2011) showed that mephedrone induces a stimulation of the dopamine transmission in the nucleus accumbens, that might be a starting point for developing drug-addiction (Volkow et al., 2003; Di Chiara et al., 2004). Although the typical dose range of MDPV appears to be between 5 and 30 mg in a single administration, some users reported tolerance with the consumption of a single dose, higher than 200 mg (Coppola and Mondola, 2012). Several users have reported a withdrawal syndrome after abrupt cessation of long-term use of methcathinone, mephedrone and MDPV (Winstock et al., 2011). Moreover, Gatch et al. (2013) showed that all of the cathinone derivatives fully substituted for methamphetamine or cocaine in drug discrimination tests. Results suggest that these drugs are comparable to cocaine and methamphetamine, and are likely to induce dependence (Iversen et al., 2014).

**Table 3:** **Studies related to the rewarding properties of amphetamine-like stimulants**.

**Substance**	**Dosage Regimen**	**Studies**	**References**
Desoxypipradrol	Rat brain slices from the nucleus accumbens core were exposed to desoxypipradrol (1, 3, or 10 μM) for 60 min	Dopamine efflux was electrically evoked and recorded using fast cyclic voltammetry. Desoxypipradrol increased the peak dopamine efflux and also slowed dopamine re-uptake. Desoxypipradrol was more potent than cocaine causing a seven fold increase in peak dopamine levels and increasing dopamine re-uptake half-life 15-fold	Davidson and Ramsey, 2011
5-APB		Voltammetric studies in rat accumbens brain slices revealed that 5-APB slowed dopamine reuptake, and at high concentrations caused reverse transport of dopamine	Dawson et al., 2014
Pentedrone	Pentedrone at 3 and 10 mg/kg significantly increased conditioned place preference in mice, while pentedrone at 0.3 mg/kg/infusion significantly increased self-administration in rats	Pentedrone produces CPP in mice and self-administration in rats. These results demonstrate the abuse liability of pentedrone in both models	Hwang et al., 2015
MDPV	Rats were trained to intravenously self-administer MDPV in daily 2 hr sessions for 10 days at doses of 0.05, 0.1, or 0.2 mg/kg/infusion	MDPV has reinforcing properties and activates brain reward circuitry, suggesting a potential for abuse and addiction in humans	Watterson et al., 2014
1-Benzylpiperazine	1.25, 5, and 20 mg/kg	1-benzylpiperazine induced place preference in the rat, which indicates that the compound possesses rewarding properties	Meririnne et al., 2006
Methamphetamine	Intravenous infusions of methamphetamine (0.15 mg/kg) in human volunteers	Intravenous methamphetamine administration produces activity in reward- and affect-related areas of the human brain including the medial orbitofrontal cortex, the rostral anterior cingulate cortex and the (ventral) striatum	Völlm et al., 2004
Mephedrone	Mephedrone was quantified between 96 and 155 mg in each tablet	Mephedrone induced strong feelings of craving in most users	Brunt et al., 2011
Mephedrone	MMC was self-administered via the intravenous route. MMC 0.1/1 mg/kg/ infusion, METH 0.01/0.3 mg/kg/ infusion	METH, but not MMC, self-administration elevated TSPO (inflammation marker translocator protein) receptor density in the nucleus accumbens and hippocampus, while MMC, but not METH,self-administration decreased striatal 5-hydroxyindolacetic acid (5-HIAA) concentrations	Motbey et al., 2013
*R*-mephedrone (*R*-MEPH) *S*-mephedrone (*S*-MEPH)	Saline, *R*-MEPH or *S*-MEPH was given for 7 days using the following doses: day 1 (15 mg/kg *R*-MEPH/*S*-MEPH or saline), days 2–6 (30 mg/kg *R*-MEPH/*S*-MEPH or saline), day 7 (15 mg/kg *R*-MEPH/*S*-MEPH or saline) Following 10 days of drug abstinence, all groups were injected with 15 mg/kg*R*-MEPH	Stereospecific effects of MEPH enantiomers suggest that the predominant dopaminergic actions of *R*-MEPH (i.e., the lack of serotonergic actions) render this stereoisomer more stimulant-like when compared with *S*-MEPH	Gregg et al., 2015
Amphetamine Mephedrone Methylone MDPV	All drugs were dissolved in physiological saline and administered (i.p.) at doses of 0.5, 2, 5, 10 or 20 mg/kg	Mephedrone, methylone and MDPV produce CPP equal or higher than amphetamine strongly suggesting addictive properties	Karlsson et al., 2014
Mephedrone Methylenedioxymethamphetamine Methamphetamine Methcathinone	4-10 or 25 mg/kg s.c.per injection, 2-h intervals, administered in a pattern used frequently to mimic psychostimulant “binge” treatment	Results revealed that, repeated mephedrone injections cause a rapid decrease in striatal dopamine (DA) and hippocampal serotonin (5-hydroxytryptamine; 5HT) transporter function. Mephedrone also inhibited both synaptosomal DA and 5HT uptake. Like methylenedioxymethamphetamine, but unlike methamphetamine or methcathinone, repeated mephedrone administrations also caused persistent serotonergic, but not dopaminergic, deficits. However, mephedrone caused DA release from a striatal suspension approaching that of methamphetamine	Hadlock et al., 2011
Mephedrone	Motor activity experiments: rats were injected with mephedrone (0.5, 1, 3, 5, 10, 30 mg/kg); CPP experiments: animals received two conditioning sessions per day, one with an injection of mephedrone (3, 10, 30 mg/kg) and the other with an injection of saline	In conclusion, mephedrone displayed locomotor stimulant properties that were dependent on increased dopamine transmission and place conditioning effects that were suggestive of rewarding properties. Those behavioral findings correlate well with neurochemical studies demonstrating that mephedrone acts as a substrate for plasma membrane monoamine transporters, evokes transporter mediated-release of monoamines through reversal of normal transporter flux, and enhances extracellular levels of dopamine and serotonin in the rat nucleus accumbens	Lisek et al., 2012
Methcathinone	Methcathinone (0.1–1.0 mg/kg),	All compounds facilitated ICSS (intracranial self-stimulation) at some doses and pretreatment times, which is consistent with abuse liability for each of these compounds. However, efficacies of compounds to facilitate ICSS varied, with methcathinone displaying the highest efficacy and mephedrone the lowest efficacy to facilitate ICSS	Bonano et al., 2014
MDPV	MDPV (0.32–3.2 mg/kg),		
Methylone	Methylone (1.0–10 mg/kg)		
Mephedrone	Mephedrone (1.0–10 mg/kg)		
MDPV alpha-PVP	Self-administration: Separate groups of rats were trained to selfadminister MDPV (*N* = 18; 0.05 mg/kg/infusion) or alpha-PVP (*N* = 9; 0.1 mg/kg/infusion, *N* = 18; 0.05 mg/kg/infusion). Telemetry procedure: Seven treatment conditions (Veh; 1, 5.6, and 10 mg/kg of alpha-PVP and MDPV) were counterbalanced and drugs were injected i.p. (1.0 ml/kg volume) with a 3–4-day interval between sessions	The potency and efficacy of MDPV and alpha-PVP were very similar across multiple assays, predicting that the abuse liability of alpha-PVP will be significant and similar to that of MDPV	Aarde et al., 2015
Methylone MDPV Mephedrone Methamphetamine	Mice were treated with methylone (30 mg/kg), MDPV (30 mg/kg), or mephedrone (40 mg/kg) using a binge-like regimen comprised four injections with a 2-h interval between each injection. For combination treatment of mice with methylone or MDPV with methamphetamine, mice were treated with varying doses of either bketoamphetamine (49 – 10, 20, or 30 mg/kg) concurrent each injection of varying doses of methamphetamine (49 – 2.5, 5, or 10 mg/kg). To determine if MDPV neuroprotection would extend to non-amphetamine neurotoxins, mice were treated with MDPV (29 – 10 mg/kg) prior to each of two injections of MPTP (20 mg/kg). All injections were given via the i.p. route	The b-ketoamphetamines alone or in all possible two-drug combinations do not result in damage to DA nerve endings but do cause hyperthermia. MDPV completely protects against the neurotoxic effects of ethamphetamine while methylone accentuates it. Neither MDPV nor methylone attenuates the hyperthermic effects of methamphetamine. The potent neuroprotective effects of MDPV extend to amphetamine-, 3,4-methylenedioxymethamphetamine-, and MPTP-induced neurotoxicity. These results indicate that b-ketoamphetamine drugs that are non-substrate blockers of the DA transporter (i.e., MDPV) protect against methamphetamine neurotoxicity, whereas those that are substrates for uptake by the DA transporter and which cause DA release (i.e., methylone, mephedrone) accentuate neurotoxicity	Anneken et al., 2015
MDPV Methylone	Self-administration studies in Rats: initial acquisition doses were 0.03 mg/kg/inj for MDPV, 0.3 or 0.5 mg/kg/inj for methylone, and 0.5 mg/kg/inj for cocaine. Microdialysis studies in Rats: drugs were administered i.v.to mimic the selfadministration route. For MDPV, rats received 0.1 mg/kg followed by 0.3 mg/kg. For methylone, rats received 1.0 mg/kg followed by 3 mg/kg	This study support the hypothesis that elevations in extracellular 5-HT in the brain can dampen positive reinforcing effects of cathinone-type drugs. Nevertheless, MDPV and methylone are both self-administered by rats, suggesting these drugs possess significant abuse liability in humans	Schindler et al., 2015
Methylone	Rats were randomly assigned to one of four groups based upon methylone dose (0.05, 0.1, 0.2, or 0.5 mg/kg per infusion)	This study reveal that methylone may possess an addiction potential similar to or greater than MDMA, yet patterns of self-administration and effects on brain reward function suggest that this drug may have a lower potential for abuse and compulsive use than prototypical psychostimulants	Watterson et al., 2012
Mephedrone Methylone MDMA	Groups of female Wistar rats were trained to self-administer mephedrone, methylone or MDMA (0.5 mg/kg/inf) under a Fixed-Ratio (FR) 1 schedule of reinforcement for 14 sessions. Following the acquisition interval, animals were evaluated in FR (0.0, 0.125, 0.25, 0.5, 1.0, 2.5 mg/kg/inf) and Progressive-ratio- PR (0.125, 1.0 mg/kg/inf) dose-substitution procedures	The results show that female rats acquired the self-administration of all three compounds with intakes in mephedrone-trained rats that were significantly higher than that of methylone-trained or MDMA-trained rats. In doses substitution under either FR or PR contingencies, however, the potencies of all three drugs were similar within the original training groups. The mephedrone-trained animals exhibited higher intakes of all drugs during dose-substitution, indicating lasting consequences of the training drug. Abuse liability of these three compounds is therefore predicted to be similar in established stimulant users but may differ in liability if they are primary drugs of initiation	Creehan et al., 2015
Mephedrone	Mephedrone (1 or 3 mg/kg)	The neurochemical and functional properties of mephedrone resemble those of MDMA, but it also shows an amphetamine-like effect in that it evokes a rapid release and elimination of DA in the brain reward system, a feature that may contribute to its potent re-inforcing properties	Kehr et al., 2011
(+)-amphetamine MDMA	MDMA (3 mg/kg) (+)-amphetamine (1 mg/kg)		

## Synthetic marijuana and the cannabimimetics

### Spice and CB1 “super agonists”

*Synthetic Cannabimimetic agents* (SC), also known as *Cannabimimetics*, are substances with pharmacological properties similar to delta-9-tetrahydrocannabinol (Δ^9^-THC) assessed by *in vitro* and *in vivo* animal studies such as binding studies and functional assays (Compton et al., 1992; EMCDDA, 2009b). SC have been detected in “Spice,” “K2,” and spice-like samples all over the world. Spice is a smokable herbal mixture marketed as a safe, legal alternative to Cannabis, composed by shredded plant material laced with a variety of SC compounds [NIDA (National Institute on Drug Abuse), 2012]. These compounds are “smokable” since they are small (typically 20–26 carbon atoms) and highly lipophilic molecules. A few hundred of SC of the JWH, HU, and CP series are currently available. They retain very high cannabinoid receptor binding affinity levels, with a dose-response efficacy significantly higher than Δ^9^-THC itself (Brents et al., 2011; Fattore and Fratta, 2011; Schifano et al., 2015). New legal regulations have been enacted to control the global diffusion of Spice. As a consequence of that, three subsequent generations of SC have been developed based on slight modifications of the first generation compounds such as JWH-018, CP 47,497, and HU-210 [ACMD (Advisory Council on the Misuse of Drugs), 2009] that are full CB1 agonists with affinities that are 4.5, 8.6, and 55 times that of Δ^9^-THC, respectively.

Different European countries, in 2009, and some states in the US, in 2010, banned the sale and use of first generation SC. These regulations induced an extreme reduction of these SC in the Spice/K2 preparations with a subsequent increase of newly synthetized SC, thus belonging to the “second” (e.g., AM-2201, MAM 2201, AM-694, RCS-4) and “third” (e.g., PB-22 “QUPIC,” 5F-PB-22, BB-22 “QUCHIC,” AB-PINACA) generation [ACMD (Advisory Council on the Misuse of Drugs), 2012, 2014] in order to avoid detection.

Several studies show that SC are remarkably different from and more dangerous than THC. Indeed, while THC is a partial CB1 agonist, *in vitro* studies have clearly shown that these compounds are full agonists with higher potency and efficacy as compared to Δ^9^-THC (Atwood et al., 2010, 2011; Marshell et al., 2014). More recent studies have been shown that selected third generation compounds, such as 5F-PB-22 and BB-22, retain greater CB1 receptor agonist potency (five- and seven- fold, respectively) and efficacy and a higher binding affinity (26- and 30-fold, respectively) at CB1 receptors compared to JWH-018 (De Luca et al., 2015b).

Moreover, studies performed in rats and mice showed that many SC displayed locomotor depressant effects and a characteristic tetrad profile at lower doses compared to Δ^9^-THC (Chaperon and Thiébot, 1999; Wiley et al., 2012, 2014; Gatch and Forster, 2014, 2015; Vigolo et al., 2015). In addition, JWH-018 and its congeners are readily metabolized to a series of cannabimimetics (Seely et al., 2012). That, together with the presence of several different SC in Spice/K2 products and their unpredictable dosing when consumed (Kronstrand et al., 2014), might explain their acute severe toxicity and even lethal medical complications in humans (Brents et al., 2011; Papanti et al., 2013; Brents and Prather, 2014; Brewer and Collins, 2014; Santacroce et al., 2015), leading to severe withdrawal syndrome and dependence as well in some cases (Zimmermann et al., 2009; Gunderson et al., 2012; Macfarlane and Christie, 2015). In addition, clinical evidence indicates that JWH-018 can generate/cause psychosis in vulnerable individuals (Every-Palmer, 2011). Notably, SC misuse has been associated with anxiety, agitation/panic attacks, paranoid ideation, suicidal ideation, and hallucinations (Fattore and Fratta, 2011; Wells and Ott, 2011; Thomas et al., 2012; Besli et al., 2015), and also been related to mood, cognitive (i.e., memory impairment, attention difficulties), neurological (i.e., dizziness, sensation changes, seizures, tremor) and psychotic (i.e., agitation, aggression, catatonia, paranoia, hallucinations, depersonalization, dissociation, prolonged psychosis, perceptual alterations) episodes, with a higher incidence in comparison to those seen with Δ^9^-THC use (Papanti et al., 2013; Spaderna et al., 2013; Van Amsterdam et al., 2015).

### Rewarding and reinforcing properties of cannabimimetics

Recent literature shows that SC have emerged as new drugs of abuse. As previously reported, an incredibly huge number of SC have been detected in Marijuana substitutes (Denooz et al., 2013; Brents and Prather, 2014; Maxwell, 2014). Being CB1 receptor agonists with extremely high affinity, SC probably act in brain regions where CB1 receptors are heavily expressed, such as the amygdala, cingulate cortex, prefrontal cortex (PFC), ventral pallidum, caudate putamen, nucleus accumbens (NAc), ventral tegmental area (VTA), and lateral hypothalamus (Glass et al., 1997; Wang et al., 2003). All these brain regions have a recognized involvement in reward, addiction and cognitive functions (Koob and Volkow, 2010). Furthermore, CB1 receptors are located in limbic regions, such as VTA, NAc, ventral pallidum, CeA, BNST, and PFC (Herkenham et al., 1991; Glass et al., 1997; Wang et al., 2003); the integration of excitatory and inhibitory inputs, coming from these structures, influence, and modulate reward processing (Sidhpura and Parsons, 2011; Panagis et al., 2014). Several studies in mice and rats showed that these compounds affect the mesolimbic dopaminergic transmission and influence conditioned behaviors (Table 4). Similar to other drugs of abuse, THC activates dopamine (DA) transmission in the ventral striatum in humans (Volkow et al., 2003; Bossong et al., 2009). In addition, animal studies showed that both Δ^9^-THC and WIN 55.212-2, a CB1 and CB2 agonist, elicit dopamine release in the NAc (Chen et al., 1993; Cheer et al., 2004) with a specific activation of the NAc shell subregion (Tanda et al., 1997; Lecca et al., 2006; De Luca et al., 2012). The NAc plays a crucial role in brain reward circuits involved in motivational and cognitive functions (Heimer et al., 1991; Zahm and Brog, 1992). In particular, it has been shown that stimulation of DA transmission in the NAc shell is directly involved in the rewarding properties of both natural reward and addictive drugs (Di Chiara et al., 2004). Microdialysis studies on awake freely moving animals performed in our laboratories showed that JWH-018, at the dose of 0.25 mg/kg i.p., increases DA transmission in the NAc shell but not in the NAc core nor in mPFC (**Figure 4**). Surprisingly, DA transmission in the NAc shell was not stimulated after administration of lower (0.125 mg/kg ip) or higher (0.5 mg/kg ip) doses producing an inverted U-shape dose response curve for the effect of JWH-018 (De Luca et al., 2015a). Further studies in mice and rats showed a similar effect after the intraperitoneal administration of JWH-073 and JWH-250 as well (Ossato et al., 2016), and after the intravenous administration of BB-22 (De Luca et al., 2015b). Notably, as previously reported by De Luca et al. (2012), THC stimulated extracellular DA release in the NAc shell at a dose fourfold higher than JWH-018 when administered intraperitoneally. In addition, BB-22 stimulates NAc shell DA release at the dose of 0.01 mg/kg iv, while THC increases extracellular DA in the same area at dose of 0.15 mg/kg iv (Tanda et al., 1997). These results show that both JWH-018 and BB-22 are more potent than THC in inducing NAc shell DA release, suggesting a putative higher abuse liability of synthetic vs. natural cannabinoids. Electrophysiological studies show that the stimulation of DA extracellular levels in the NAc shell by JWH-018 is thought to be due to the activation of CB1 receptors located on presynaptic GABAergic afferents directed to VTA DA neurons, leading to a reduction of GABA_A_ receptors mediated inhibition of DA neuronal activity in the VTA (Lupica and Riegel, 2005; Mátyás et al., 2008; Melis et al., 2014; De Luca et al., 2015a). Rewarding effects of cannabimimetics have also been assessed by different experimental paradigms such as intracranial self-stimulation (ICSS), place conditioning tests, drug-discrimination and intravenous self-administration (IVSA) studies. ICSS of the medial forebrain bundle is the operant conditioning method used in rodents to evaluate the role of the mesolimbic dopamine pathway in rewarding behavioral effects (Carlezon and Chartoff, 2007) and evaluating potential of abuse (Negus and Miller, 2014). Not surprisingly, to date no data on the effect of new SC on ICSS are available. Δ^9^-THC does not facilitate ICSS, but has a dose-dependent inhibitory influence on ICSS (Vlachou et al., 2007). Similarly, a depression of ICSS is observed after the administration of WIN55212-2, CP55940, HU210 (Antoniou et al., 2005; Vlachou et al., 2005; Mavrikaki et al., 2010). Differences in developing tolerance to depression of ICSS after repeated exposure to cannabinoids have been reported. Tolerance is completely developed after repeated exposure to Δ^9^-THC (Kwilasz and Negus, 2012) but partially developed after CP55940 (Grim et al., 2015), and not developed after WIN55212-2 administration (Mavrikaki et al., 2010), suggesting that the different affinity of Δ^9^-THC vs. SC for the CB1 receptors could play a role in developing this tolerance (Grim et al., 2015).

**Table 4:** **Studies related to the rewarding properties of cannabimimetics**.

**Substance**	**Dosage Regimen**	**Studies**	**References**
WIN 55212-2	Intravenous self-administration model in drug-naive mice of WIN 55212-2 (0.5 and 0.1 mg/kg per injection)	WIN 55,212-2 was intravenously self-administered by mice in a concentration-dependent manner according to a bell-shaped curve	Martellotta et al., 1998
HU210	Conditioned place preference (CPP) in male rats: HU210 (20, 60 and 100 μg/kg), and Δ^9^-THC (1.5 mg/kg)	HU210 and Δ^9^-THC produced aversion as expressed by time spent in the drug-paired compartment of the CPP apparatus	Cheer et al., 2000
WIN 55212-2	Intravenous SA in rats WIN 55,212-2 at doses ranging from 6.25 to 50 μg/kg per injection, under a fixed-ratio 1 (FR1) schedule of reinforcement and nose-pokes as the operant responses	Response rate depended on the drug dose available, with maximum rates occurring at 12.5 microg/kg per injection	Fattore et al., 2001
WIN 55212-2	Fast-scan cyclic voltammetry: systemic administration at a dose of 125 μg/kg	WIN55,212–2 enhances dopamine transients but depresses electrically evoked release	Cheer et al., 2004
WIN 55212-2 CP 55940 HU-210	After Intracranial self-stimulation (ICSS) of the medial forebrain bundle, rats received intraperitoneal injections of WIN 55,212-2 (graded doses 0.1, 0.3, 1 and 3 mg/kg), CP 55,940 (graded doses 10, 30, 56 and 100 μg/kg), or HU-210 (graded doses 10, 30, 100 μg/kg)	With the exception of the highest dose of all cannabinoid agonists tested, which significantly increased the threshold frequency required for ICSS into the medial forebrain bundle, all other doses of the tested drugs did not affect ICSS thresholds. The CB1 receptor antagonist SR141716A reversed the actions of WIN 55,212-2 and CP 55,940, but not HU-210	Vlachou et al., 2005
WIN 55212-2	Intravenous self-administration (SA). Rats, trained for 3 weeks to self-administer WIN 55,212-2 (12.5 μg/kg) in single daily 1-h sessions under a fixed ratio 1 (FR 1) schedule, then switched to FR 2 for a further week. During SA sessions, microdialysis assays were performed every 3rd day, and then daily starting from the 13th session. Dialysate DA from the NAc shell and core was monitored before, during, and for 30 min after SA	Response-contingent WIN 55,212-2 SA preferentially increases the NAc shell DA output as compared to that of the core independently from the duration of the WIN 55,212-2 exposure. Increase in NAc DA is strictly related to WIN 55,212-2 actions because it is not observed during extinction despite active responding	Lecca et al., 2006
WIN 55212-2	Rats received intraperitoneal injections of WIN55,212-2 (0.1, 0.3 or 1 mg/kg) for 20 subsequent days. Thresholds for ICSS were measured before and after each injection	WIN55,212-2 (1 mg/kg) significantly increased ICSS thresholds from the first day of administration, an effect that remained stable across the subsequent days of administration. These findings indicate that repeated WIN55,212-2 administration elicited a sustained increase in ICSS	Mavrikaki et al., 2010
JWH-018 JWH-073 JWH-210	Adult male rats trained to discriminate 3 mg/kg Δ(9)-THC or 0.3 mg/kg JWH-018 from vehicle	JWH-018, JWH-073, and JWH-210 fully substituted in Δ(9)-THC-trained rats and Δ(9)-THC substituted in JWH-018-trained rats	Wiley et al., 2014
JWH-018 JWH-073 JWH-250 JWH-200 JWH-203 AM-2201 CP 47,497-C8-homolog	These compounds were then tested for substitution in rats trained to discriminate Δ-THC (3 mg/kg, intraperitoneally)	Each of the compounds fully substituted for the discriminative stimulus effects of Δ-THC, mostly at doses that produced only marginal amounts of rate suppression. JWH-250 and CP 47,497-C8-homolog suppressed response rates at doses that fully substituted for Δ-THC	Gatch and Forster, 2014
CP 55940	Acute and repeated administration (7 days) of CP55,940 (0.12-0.18)mg/kg).on operant responding for electrical brain stimulation of the medial forebrain bundle in C57BL/6J mice	CP55,940 attenuated ICSS in a dose-related manner. This effect was blocked by the CB1 receptor antagonist rimonabant	Grim et al., 2015
JWH-018	Microdialysis studies in rats: 0.125 mg/kg ip 0. 25 mg/kg ip 0. 5 mg/kg ip Rats self-administered JWH-018 (20 μg/kg/infusion) in single daily 1 h FR3 sessions. C57BL/6 mice self-administered JWH-018 (30 μg/kg/infusion) in single daily 2 h FR1 sessions	JWH-018 0.25 mg/kg ip increases dopamine transmission in Nac shell, but not in NAc core nor in mPFC. The lower and the higher doses do not stimulate DA transmission so the dose-response curve of this compound has an inverted U-shape. Both rats and mice readily acquired two different operant behaviors: nose-poking into an optical switch (rats) and lever-pressing (mice)	De Luca et al., 2015a
BB-22 5F-PB-22 5F-AKB-48 STS-135	Microdialysis studies in rats: BB-22 (0.003-0.01 mg/kg i.v.) 5F-PB-22 (0.01 mg/kg i.v.) 5F-AKB-48 (0.1 mg/kg i.v.) STS-135(0.15 mg/kg i.v.)	BB-22 (0.003-0.01 mg/kg i.v.) increased dialysate DA in the accumbens shell but not in the core or in the medial prefrontal cortex, with bell shaped dose-response curve and an effect at 0.01 mg/kg and a biphasic time-course; systemic AM251 (1.0 mg/kg i.p.) completely prevented the stimulant effect of BB-22 on dialysate DA in the NAc shell. All the other compounds increased dialysate DA in the NAc shell at doses consistent with their *in vitro* affinity	De Luca et al., 2015b

On the other hand, place conditioning tests in animals showed that WIN 55212-2 and HU210 established a robust place aversion (CPA), reversed by the CB1 receptor antagonist/inverse agonist SR 141716A, in a similar way as seen with Δ^9^-THC (Chaperon et al., 1998; Cheer et al., 2000; Valjent and Maldonado, 2000). In addition, the CB1 antagonist/inverse agonist AM281 did not induce conditioned place preference (CPP). However, a pre-treatment of 14 days with AM281 prior to the CPP test with the same drug, showed significant CPP (Botanas et al., 2015). Accordingly, it has been hypothesized that the endogenous cannabinoid system in the brain may act as a counter-reward system, and blocking or antagonizing this system would therefore produce the reward (Sañudo-Peña et al., 1997; Botanas et al., 2015). This could represent a limitation on the therapeutic use of CB1 antagonist/inverse agonists (Seely et al., 2011).

The psychopharmacological effects of SC have also been assessed by drug-discrimination studies. These experimental paradigms represent useful tools for evaluating the abuse liability of new drugs that might produce dependence (Solinas et al., 2006). Drug-discrimination studies in rats have showed that JWH-018, JWH-250 and CP 47,497-C8-homolog, UR-144, XLR-11, AKB-48 (APINACA), PB-22 (QUPIC), 5F-PB-22, and AB-FUBINACA fully substituted for the discriminative stimulus effects of Δ^9^-THC (Gatch and Forster, 2014, 2015; Wiley et al., 2014).

These studies typically serve as an integration of the results obtained by intravenous self-administration (SA) experiments. As for SA studies, while there is some disputable data concerning the reinforcing properties of Δ^9^-THC based on its ability to be persistently self-administered in squirrel monkeys (Tanda et al., 2000) but not in rodents, there is still favorable evidence available about SC SA. Thus it has been reported that monkeys, mice and rats acquire and maintain WIN 55.212-2 SA (Martellotta et al., 1998; Fattore et al., 2001; Justinova et al., 2004; Lecca et al., 2006). Recently, it has been reported that JWH-018 is self-administered in rodents (**Figure 5**). In the study by De Luca et al. (2015a) both rats and mice readily acquired two different operant behaviors: nose-poking into an optical switch (rats) and lever-pressing (mice). Rats self-administered JWH-018 at the dose of 20 μg/kg/infusion in daily 1 h FR3 sessions (**Figure 5A**). As expected, a reduction of SA after the injection of SR141617A (1 mg/kg ip, 30 min prior to the SA session) was observed, consistent with the lack of JWH-018 mediated reinforcement. Intriguingly, nose-poking for JWH-018 significantly increased from the first session (30th session, **Figure 5A**) performed after the administration of SR141617A for 2 consecutive days, confirming that these effects of JWH-018 are mediated through cannabinoid receptors. SA behavior did not decrease when JWH-018 was replaced by vehicle. A control group of rats trained for vehicle, failed to acquire SA behavior. It has been hypothesized that the absence of extinction-like response patterns was unrelated to response-contingent training for JWH-018 because the vehicle failed to induce responding. We think that this probably occurred as a result of a habit learning conditioned by JWH-018, in fact contextual cues were sufficient to maintain responding (De Luca et al., 2015a). This confirmed previous evidence showing that endocannabinoid signaling through CB1 receptors is significant for the habit formation (Hilário et al., 2007). Indeed, in mice, CB1 receptor knockdown can enhance or blunt habit formation, whereas Δ^9^-THC tolerance enhances habit formation; in humans, cannabis use enhances the stimulus-response/habit memory (for review, see Goodman and Packard, 2015).

JWH-018 self-administration studies performed in C57BL/6 mice show that animals acquired SA at the dose of 30 μg/kg/infusion in daily 2 h FR1 sessions (**Figure 5B**). Importantly, the specificity of mice responding behavior has been confirmed by the increase of SA under progressive-ratio (PR) schedule of reinforcement. During extinction phase, surprisingly, active lever-pressing did not decrease, while inactive lever-pressing increased becoming superimposable to the active ones. Unlike the SA experiments with rats, in these experiments, the drug associated cues were removed after the first three of a total of 12 sessions during the extinction phase. However, during the JWH-018 reinstatement, SA behavior was immediately reinstated and inactive lever-presses decreased since the SA behavior was specifically regulated by the drug infusion confirming the reinforcing properties of JWH-018. This may also prove that JWH-018 alters cortical processes important for the context updating and the automatic orientation of attention (D'Souza et al., 2012) with consequent disruption of cognitive functioning, emotional processing, and affective states as different SC make in humans (Zimmermann et al., 2009). Further research is needed to evaluate the impact of chronic exposure to SC.

## Concluding remarks

The review of the biomedical data here presented, clearly demonstrates the unsafe nature of these new drugs of abuse. This is particularly alarming since adolescents seem to be the most exposed subjects to these dangerous NPS. Indeed, users are often unaware of the consequences of ingesting synthetic compounds that are sold as “legal alternative” to classical drugs, and their unexpected, sometimes fatal adverse effects. Only awareness can reduce NPS use since stopping their synthesis and diffusion seems to be an improbable task and, morbidity and mortality reports keep increasing as NPS gain popularity worldwide. Awareness campaigns about these substances and their devastating effects should be organized to inform everyone, including clinicians, who should be able to recognize symptoms of intoxication induced by NPS (Simonato et al., 2013; Papanti et al., 2013; Schifano et al., 2015).

Ultimately, this paper intends to be helpful to drive governments and civil society to not underestimate the NPS issue, and to encourage the scientific community to deeply evaluate the pharmacology and toxicological effects of NPS and to develop effective treatments for NPS intoxication. Additionally, this paper intends to be useful for advising law enforcement agencies, which need updated information for the prevention and fight against trafficking and sale of NPS, and in the long run, hopefully contribute to better protect public health and safety.

## Author contributions

**Figure 3:**
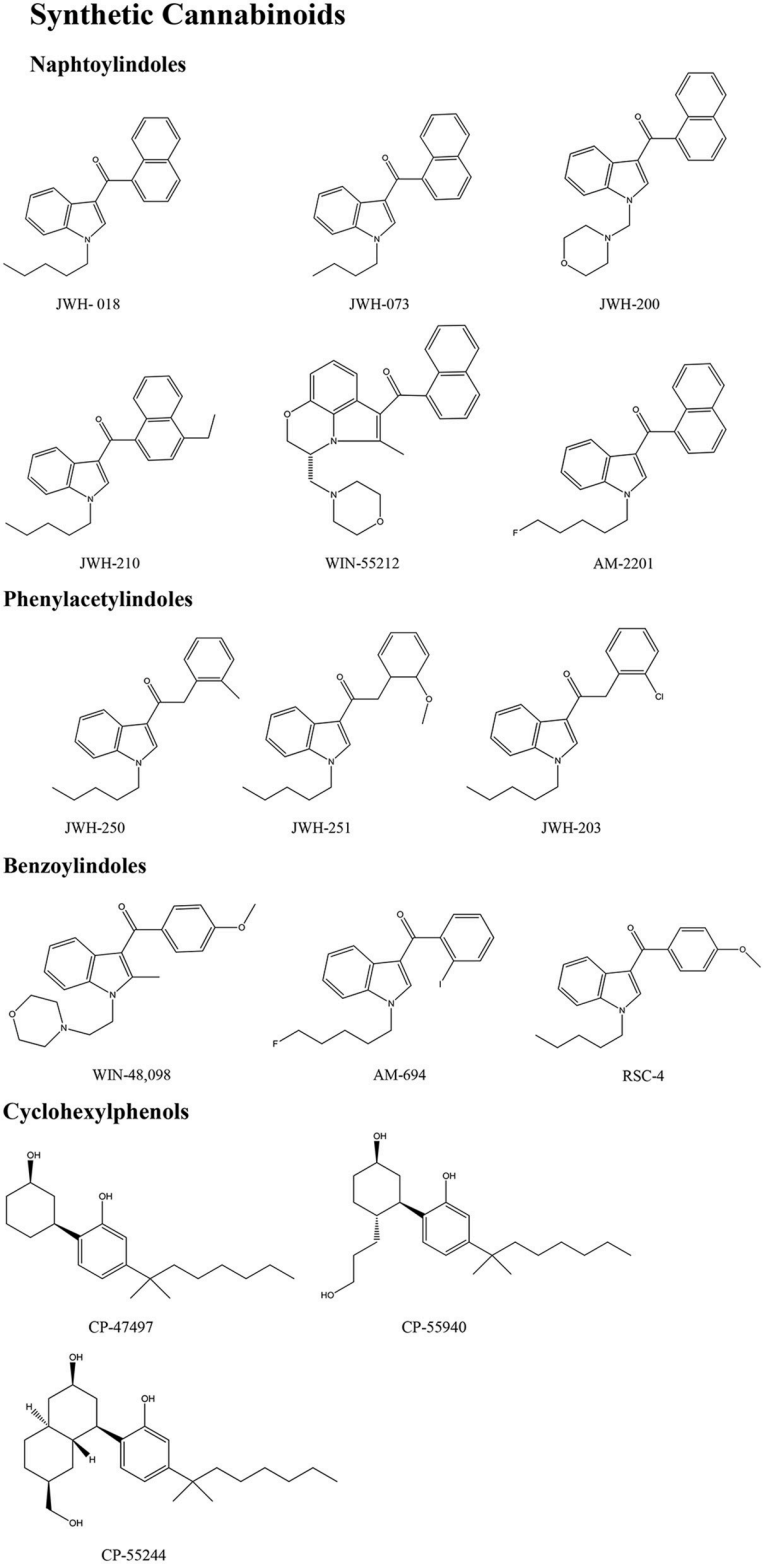
**Chemical structures of Synthetic Cannabinoids**.

**Figure 4:**
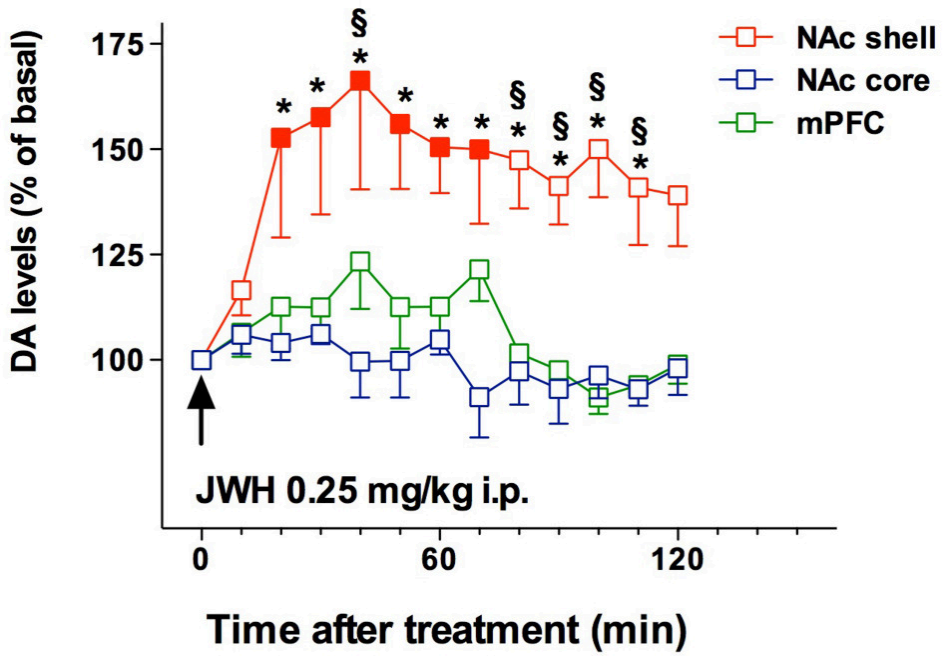
**Effect of JWH-018 administration on DA transmission in the NAc shell, NAc core, and mPFC**. Results are expressed as mean ± SEM of change in DA extracellular levels expressed as the percentage of basal values. The arrow indicates the start of JWH-018 i.p. injection at the dose of 0.25 mg/kg in the NAc shell (*red squares*), NAc core (*blue squares*), and mPFC (*green squares*). Solid symbol: *p* < 0.05 with respect to basal values; ^*^p < 0.05 vsNAc core group; § *p* < 0.05 vs mPFC group; (NAc shell *N* = 10; NAc core *N* = 7; mPFC *N* = 11) (Two-way ANOVA, Tukey's HSD post hoc). Adapted from De Luca et al. (2015a).

**Figure 5:**
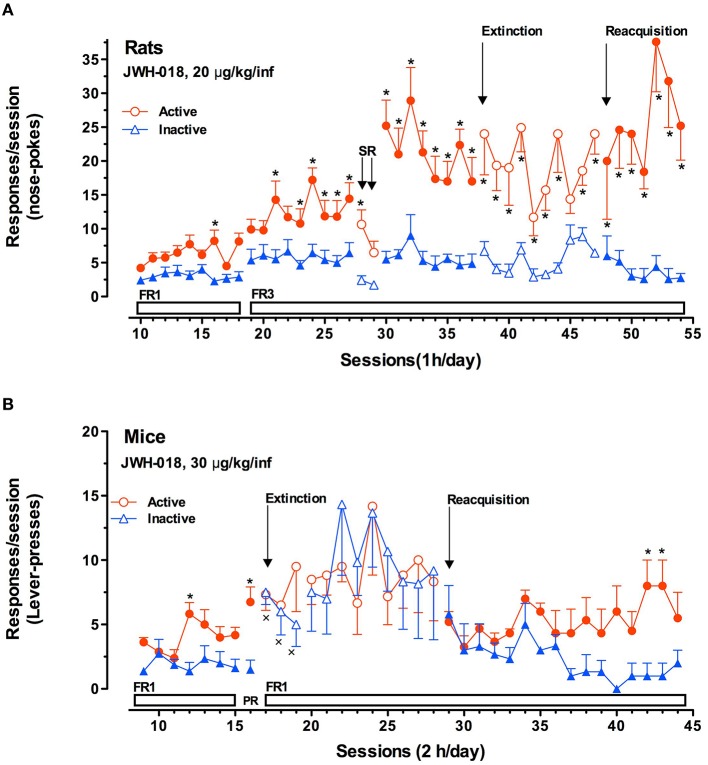
**JWH-018 self-administration in rats and mice. (A)** JWH-018 self-administration by Sprague-Dawley rats and involvement of CB1 cannabinoid receptors in this behavior. Number of active nose pokes (*circles*) that resulted in JWH-018 infusion (20 μg/kg/infusion) or inactive ones (*triangles*) during each 1-h daily session under FR1 and FR 3 during acquisition (1th to 37th sessions), extinction (38th To 47th sessions) and reacquisition (48th to 54thsessions) phases. On sessions 28th and 29th the effect of SR 141716A on the JWH-018 SA was tested. Results are expressed as mean ± SEM (N sessions 10–47 = 14, sessions 48–54 = 6) ^*^p < 0.05 vs. inactive nose pokes; ANOVA followed by LSD *post hoc* test. **(B)** JWH-018 self-administration by C57BL/6 mice under fixed (FR1) and progressive (PR) reinforcement schedules. Number of active lever-presses (*circles*) that resulted in JWH-018 infusion (30 μg/kg/inf) or inactive lever-presses (*triangles*) during each 2 h daily session under FR1 (9th–15th sessions), and PR (16th session) reinforcement schedules. Results are expressed as mean ± SEM (*N* = 8), ^*^p < 0.05 vs. inactive lever- presses; ANOVA followed by LSD *post hoc* test. Adapted from De Luca et al. (2015a).

This is a review, different authors contributed as follows: CM: Section NPS: From Chemistry to Pharmacological Effects; Figures 1–3, Tables 1, 3, 4. GS and CR: Section–Introduction; MM and MM: Section–Human and Animal Studies on amphetamine-Like Stimulant Effects: Psychoactive Effects, Cognitive Deficits, Emotional Alterations, and Dependence -MM: Table 2. MDL: Section–Synthetic Marijuana and the Cannabimimetics, Section–Concluding Remarks and entire revision of the manuscript; Figures 4, 5 and Tables 3, 4.

### Conflict of interest statement

The authors declare that the research was conducted in the absence of any commercial or financial relationships that could be construed as a potential conflict of interest.

## References

[B1] Aarde S. M. Creehan K. M. Vandewater S. A. Dickerson T. J. Taffe M. A. (2015). *In vivo* potency and efficacy of the novel cathinone α-pyrrolidinopentiophenone and 3,4-methylenedioxypyrovalerone: self-administration and locomotor stimulation in male rats. Psychopharmacology (Berl.) 232, 3045–3055. 10.1007/s00213-015-3944-8 25925780 PMC4515201

[B2] Aarde S. M. Huang P. K. Creehan K. M. Dickerson T. J. Taffe M. A. (2013). The novel recreational drug 3,4-methylenedioxypyrovalerone (MDPV) is a potent psychomotor stimulant: self-administration and locomotor activity in rats. Neuropharmacology 71, 130–140. 10.1016/j.neuropharm.2013.04.003 23597511 PMC3681807

[B3] ACMD (Advisory Council on the Misuse of Drugs) (2009). Review of the ACMD, 2009 - Publications - GOV.UK. Available online at: https://www.gov.uk/government/publications/review-of-the-acmd-2009 (Accessed October 29, 2015).

[B4] ACMD (Advisory Council on the Misuse of Drugs) (2012). Methoxetamine report, 2012 - Publications - GOV.UK. Available at: https://www.gov.uk/government/publications/advisory-council-on-the-misuse-of-drugs-acmd-methoxetamine-report-2012 (Accessed October 29, 2015).

[B5] ACMD (Advisory Council on the Misuse of Drugs) (2013). Ketamine: A Review of Use and Harm, London.

[B6] ACMD (Advisory Council on the Misuse of Drugs) (2014). IMSD 2014/ACMD 2014. Available online at: http://imsd-acmd2014.ksme.or.kr/main/ (Accessed October 29, 2015)

[B7] Anneken J. H. Angoa-Pérez M. Kuhn D. M. (2015). 3,4-Methylenedioxypyrovalerone prevents while methylone enhances methamphetamine-induced damage to dopamine nerve endings: β-ketoamphetamine modulation of neurotoxicity by the dopamine transporter. J. Neurochem. 133, 211–222. 10.1111/jnc.13048 25626880 PMC4759647

[B8] Antoniou K. Galanopoulos A. Vlachou S. Kourouli T. Nahmias V. Thermos K.. (2005). Behavioral pharmacological properties of a novel cannabinoid 1′,1′-dithiolane delta8-THC analog, AMG-3. Behav. Pharmacol. 16, 499–510. 10.1097/00008877-200509000-00024 16148456

[B9] Araújo A. M. Carvalho F. Bastos M. de L. Guedes de Pinho P. Carvalho M. (2015). The hallucinogenic world of tryptamines: an updated review. Arch. Toxicol. 89, 1151–1173. 10.1007/s00204-015-1513-x 25877327

[B10] Atwood B. K. Huffman J. Straiker A. MacKie K. (2010). JWH018, a common constituent of “Spice” herbal blends, is a potent and efficacious cannabinoid CB 1 receptor agonist. Br. J. Pharmacol. 160, 585–593. 10.1111/j.1476-5381.2009.00582.x 20100276 PMC2931559

[B11] Atwood B. K. Lee D. Straiker A. Widlanski T. S. Mackie K. (2011). CP47,497-C8 and JWH073, commonly found in “Spice” herbal blends, are potent and efficacious CB(1) cannabinoid receptor agonists. Eur. J. Pharmacol. 659, 139–145. 10.1016/j.ejphar.2011.01.066 21333643 PMC3094488

[B12] Baumann M. H. Clark R. D. Rothman R. B. (2008). Locomotor stimulation produced by 3,4-methylenedioxymethamphetamine (MDMA) is correlated with dialysate levels of serotonin and dopamine in rat brain. Pharmacol. Biochem. Behav. 90, 208–217. 10.1016/j.pbb.2008.02.018 18403002 PMC2491560

[B13] Baumann M. H. Ayestas M. A. Jr. Partilla J. S. Sink J. R. Shulgin A. T. Daley P. F.. (2012). The designer methcathinone analogs, mephedrone and methylone, are substrates for monoamine transporters in brain tissue. Neuropsychopharmacology 37, 1192–1203. 10.1038/npp.2011.304 22169943 PMC3306880

[B14] Baumann M. H. Partilla J. S. Lehner K. R. (2013). Psychoactive “bath salts”: Not so soothing. Eur. J. Pharmacol. 698, 1–5. 10.1016/j.ejphar.2012.11.020 23178799 PMC3537229

[B15] Bersani F. S. Corazza O. Albano G. Valeriani G. Santacroce R. Bolzan Mariotti Posocco F.. (2014). 25C-NBOMe: preliminary data on pharmacology, psychoactive effects, and toxicity of a new potent and dangerous hallucinogenic drug. Biomed Res. Int. 2014:734749. 10.1155/2014/734749 25105138 PMC4106087

[B16] Besli G. E. Ikiz M. A. Yildirim S. Saltik S. (2015). Synthetic cannabinoid abuse in adolescents: a case series. J. Emerg. Med. 49, 644–650. 10.1016/j.jemermed.2015.06.053 26293411

[B17] Bonano J. S. Glennon R. A. De Felice L. J. Banks M. L. Negus S. S. (2014). Abuse-related and abuse-limiting effects of methcathinone and the synthetic “bath salts” cathinone analogs methylenedioxypyrovalerone (MDPV), methylone and mephedrone on intracranial self-stimulation in rats. Psychopharmacology (Berl). 231, 199–207. 10.1007/s00213-013-3223-5 23949206 PMC3877726

[B18] Bossong M. G. van Berckel B. N. M. Boellaard R. Zuurman L. Schuit R. C. Windhorst A. D.. (2009). Delta 9-tetrahydrocannabinol induces dopamine release in the human striatum. Neuropsychopharmacology 34, 759–766. 10.1038/npp.2008.138 18754005

[B19] Botanas C. J. de la Peña J. B. Dela Pena I. J. Tampus R. Kim H. J. Yoon S. S.. (2015). Evaluation of the abuse potential of AM281, a new synthetic cannabinoid CB1 receptor antagonist. Eur. J. Pharmacol. 766, 135–141. 10.1016/j.ejphar.2015.10.004 26450088

[B20] Brents L. K. Prather P. L. (2014). The K2/Spice phenomenon: emergence, identification, legislation and metabolic characterization of synthetic cannabinoids in herbal incense products. Drug Metab. Rev. 46, 72–85. 10.3109/03602532.2013.839700 24063277 PMC4100246

[B21] Brents L. K. Reichard E. E. Zimmerman S. M. Moran J. H. Fantegrossi W. E. Prather P. L. (2011). Phase I hydroxylated metabolites of the K2 synthetic cannabinoid JWH-018 retain *in vitro* and *in vivo* cannabinoid 1 receptor affinity and activity. PLoS ONE 6:e21917. 10.1371/journal.pone.0021917 21755008 PMC3130777

[B22] Brewer T. L. Collins M. (2014). A review of clinical manifestations in adolescent and young adults after use of synthetic cannabinoids. J. Spec. Pediatr. Nurs. 19, 119–126. 10.1111/jspn.12057 24320158

[B23] Brunt T. M. Poortman A. Niesink R. J. M. van den Brink W. (2011). Instability of the ecstasy market and a new kid on the block: mephedrone. J. Psychopharmacol. 25, 1543–1547. 10.1177/0269881110378370 20826554

[B24] Burns L. Roxburgh A, Bruno, R. Van Buskirk J. (2014). Monitoring drug markets in the Internet age and the evolution of drug monitoring systems in Australia. Drug Test. Anal. 6, 840–845. 10.1002/dta.1613 24574080

[B25] Carlezon W. A. Jr. Chartoff E. H. (2007). Intracranial self-stimulation (ICSS) in rodents to study the neurobiology of motivation. Nat. Protoc. 2, 2987–2995. 10.1038/nprot.2007.441 18007634

[B26] Chaperon F. Thiébot M. H. (1999). Behavioral effects of cannabinoid agents in animals. Crit. Rev. Neurobiol. 13, 243–281. 10803637 10.1615/critrevneurobiol.v13.i3.20

[B27] Chaperon F. Soubrié P. Puech A. J. Thiébot M. H. (1998). Involvement of central cannabinoid (CB1) receptors in the establishment of place conditioning in rats. Psychopharmacology (Berl) 135, 324–332. 10.1007/s002130050518 9539255

[B28] Cheer J. F. Kendall D. A. Marsden C. A. (2000). Cannabinoid receptors and reward in the rat: a conditioned place preference study. Psychopharmacology (Berl). 151, 25–30. 10.1007/s002130000481 10958113

[B29] Cheer J. F. Wassum K. M. Heien M. L. A. V. Phillips P. E. M. Wightman R. M. (2004). Cannabinoids enhance subsecond dopamine release in the nucleus accumbens of awake rats. J. Neurosci. 24, 4393–4400. 10.1523/JNEUROSCI.0529-04.2004 15128853 PMC6729440

[B30] Chen J. Marmur R. Pulles A. Paredes W. Gardner E. L. (1993). Ventral tegmental microinjection of delta 9-tetrahydrocannabinol enhances ventral tegmental somatodendritic dopamine levels but not forebrain dopamine levels: evidence for local neural action by marijuana's psychoactive ingredient. Brain Res. 621, 65–70. 10.1016/0006-8993(93)90298-2 8221074

[B31] Compton D. R. Johnson M. R. Melvin L. S. Martin B. R. (1992). Pharmacological profile of a series of bicyclic cannabinoid analogs: classification as cannabimimetic agents. J. Pharmacol. Exp. Ther. 260, 201–209. 1309872

[B32] Coppola M. Mondola R. (2012). Synthetic cathinones: chemistry, pharmacology and toxicology of a new class of designer drugs of abuse marketed as “bath salts” or “plant food.” Toxicol. Lett. 211, 144–149. 10.1016/j.toxlet.2012.03.009 22459606

[B33] Corazza O. Schifano F. Farre M. Deluca P. Davey Z. Torrens M.. (2011). Designer drugs on the internet: a phenomenon out-of-control? the emergence ofhallucinogenic drug Bromo-Dragonfly. Curr. Clin. Pharmacol. 6, 125–129. 10.2174/157488411796151129 21592070

[B34] Corazza O. Valeriani G. Bersani F. S. Corkery J. Martinotti G. Bersani G.. (2014). “Spice,” “kryptonite,” “black mamba”: an overview of brand names and marketing strategies of novel psychoactive substances on the web. J. Psychoactive Drugs 46, 287–294. 10.1080/02791072.2014.944291 25188698

[B35] Corkery J. M. Schifano F. Ghodse A. H. (2012). Mephedrone-related fatalities in the United Kingdom: contextual, clinical and practicalissues, in *Pharmacology,* ed Gallelli L. (Rijeka: InTech), 355–380.

[B36] Corkery J. M. Elliott S. Schifano F. Corazza O. Ghodse A. H. (2013). MDAI (5,6-methylenedioxy-2-aminoindane; 6,7-dihydro-5H-cyclopenta[f][1,3]benzodioxol-6-amine; “sparkle”; “mindy”) toxicity: a brief overview and update. Hum. Psychopharmacol. Clin. Exp. 28, 345–355. 10.1002/hup.2298 23881883

[B37] Corkery J. M. Claridge H. Loi B. Goodair C. Schifano F. (2014). Drug Related Deaths in the, UK. NPSAD Annual Report 2013. London: International Centre for Drug Policy; St. George's University of London.

[B38] Cozzi N. V. Gopalakrishnan A. Anderson L. L. Feih J. T. Shulgin A. T. Daley P. F.. (2009). Dimethyltryptamine and other hallucinogenic tryptamines exhibit substrate behavior at the serotonin uptake transporter and the vesicle monoamine transporter. J. Neural Transm. 116, 1591–1599. 10.1007/s00702-009-0308-8 19756361

[B39] Creehan K. M. Vandewater S. A. Taffe M. A. (2015). Intravenous self-administration of mephedrone, methylone and MDMA in female rats. Neuropharmacology 92, 90–97. 10.1016/j.neuropharm.2015.01.003 25600245 PMC4346510

[B40] Davidson C. Ramsey J. (2011). Desoxypipradrol is more potent than cocaine on evoked dopamine efflux in the nucleus accumbens. J. Psychopharmacol. 26, 1036–1041. 10.1177/0269881111430733 22158543

[B41] Dawson P. Opacka-Juffry J. Moffatt J. D. Daniju Y. Dutta N. Ramsey J.. (2014). The effects of benzofury (5-APB) on the dopamine transporter and 5-HT2-dependent vasoconstriction in the rat. Prog. Neuro Psychopharmacol. Biol. Psychiatry 48, 57–63. 10.1016/j.pnpbp.2013.08.013 24012617

[B42] De Luca M. A. Bimpisidis Z. Melis M. Marti M. Caboni P. Valentini V.. (2015a). Stimulation of *in vivo* dopamine transmission and intravenous self-administration in rats and mice by JWH-018, a Spice cannabinoid. Neuropharmacology 99, 705–714. 10.1016/j.neuropharm.2015.08.041 26327678

[B43] De Luca M. A. Solinas M. Bimpisidis Z. Goldberg S. R. Di Chiara G. (2012). Cannabinoid facilitation of behavioral and biochemical hedonic taste responses. Neuropharmacology 63, 161–168. 10.1016/j.neuropharm.2011.10.018 22063718 PMC3705914

[B44] De Luca M. A. Castelli M. P. Loi B. Porcu A. Martorelli M. Miliano C.. (2015b). Native CB1 receptor affinity, intrisic activity and accumbens shell dopamine stimulant properties of third generation spice/K2 cannabinoids: BB-22, 5F-PB-22, 5F-AKB-48 and STS-135. Neuropharmacology. 105, 630–638. 10.1016/j.neuropharm.2015.11.017 26686391

[B45] Dean B. V. Stellpflug S. J. Burnett A. M. Engebretsen K. M. (2013). 2C or not 2C: phenethylamine designer drug review. J. Med. Toxicol. 9, 172–178. 10.1007/s13181-013-0295-x 23494844 PMC3657019

[B46] Deluca P. Davey Z. Corazza O. Di Furia L. Farre M. Flesland L. H.. (2012). Identifying emerging trends in recreational drug use; outcomes from the Psychonaut Web Mapping Project. Prog. Neuro Psychopharmacol. Biol. Psychiatry 39, 221–226. 10.1016/j.pnpbp.2012.07.011 22841965

[B47] Denooz R. Vanheugen J.-C. Frederich M. de Tullio P. Charlier C. (2013). Identification and structural elucidation of four cannabimimetic compounds (RCS-4, AM-2201, JWH-203 and JWH-210) in seized products. J. Anal. Toxicol. 37, 56–63. 10.1093/jat/bks095 23339188

[B48] Di Chiara G. Bassareo V. Fenu S. De Luca M. A. Spina L. Cadoni C.. (2004). Dopamine and drug addiction: The nucleus accumbens shell connection. Neuropharmacology 47, 227–241. 10.1016/j.neuropharm.2004.06.032 15464140

[B49] Drug Policy Department Italian Presidency of the Council of Ministers (2013a). National Action Plan on New Psychoactive Substances, Rome.

[B50] Drug Policy Department Italian Presidency of the Council of Ministers (2013b). New Psychoactive Substances. Rome.

[B51] Drug Policy Department Italian Presidency of the Council of Ministers (2014). Annual Report on Drug. Rome.

[B52] D'Souza D. C. Fridberg D. J. Skosnik P. D. Williams A. Roach B. Singh N.. (2012). Dose-related modulation of event-related potentials to novel and target stimuli by intravenous delta^9^-THC in humans. Neuropsychopharmacology 37, 1632–1646. 10.1038/npp.2012.8 22334121 PMC3358754

[B53] EMCDDA (European Drug Report) (2014). Trends and Developments. Available online at: http://www.emcdda.europa.eu/publications/edr/trends-developments/2014 (Accessed November 14, 2015).

[B54] EMCDDA (2009a). Annual Report on the State of the Drugs Problem in Europe. Available online at: http://www.emcdda.europa.eu/publications/annual-report/2009 (Accessed October 29, 2015).

[B55] EMCDDA (2009b). Thematic Papers. Understanding the “Spice” phenomenon.

[B56] EMCDDA (European Drug Report) (2015a). Trends and Developments. Available online at: http://www.emcdda.europa.eu/publications/edr/trends-developments/2015 (Accessed November 14, 2015).

[B57] EMCDDA (New psychoactive substances in Europe) (2015b). An Update from the EU Early Warning System. Available online at: http://www.emcdda.europa.eu/publications/2015/new-psychoactive-substances (Accessed December 14, 2015).

[B58] Eshleman A. J. Forster M. J. Wolfrum K. M. Johnson R. A. Janowsky A. Gatch M. B. (2014). Behavioral and neurochemical pharmacology of six psychoactive substituted phenethylamines: mouse locomotion, rat drug discrimination and *in vitro* receptor and transporter binding and function. Psychopharmacology (Berl.) 231, 875–888. 10.1007/s00213-013-3303-6 24142203 PMC3945162

[B59] Eshleman A. J. Wolfrum K. M. Hatfield M. G. Johnson R. A. Murphy K. V. Janowsky A. (2013). Substituted methcathinones differ in transporter and receptor interactions. Biochem. Pharmacol. 85, 1803–1815. 10.1016/j.bcp.2013.04.004 23583454 PMC3692398

[B60] Every-Palmer S. (2011). Synthetic cannabinoid JWH-018 and psychosis: an explorative study. Drug Alcohol Depend. 117, 152–157. 10.1016/j.drugalcdep.2011.01.012 21316162

[B61] Fantegrossi W. E. Murnane K. S. Reissig C. J. (2008). The behavioral pharmacology of hallucinogens. Biochem. Pharmacol. 75, 17–33. 10.1016/j.bcp.2007.07.018 17977517 PMC2247373

[B62] Fass J. A. Fass A. D. Garcia A. S. (2012). Synthetic Cathinones (Bath Salts): legal status and patterns of abuse. Ann. Pharmacother. 46, 436–441. 10.1345/aph.1Q628 22388331

[B63] Fattore L. Fratta W. (2011). Beyond THC: The new generation of cannabinoid designer drugs. Front. Behav. Neurosci. 5:60. 10.3389/fnbeh.2011.00060 22007163 PMC3187647

[B64] Fattore L. Cossu G. Martellotta C. M. Fratta W. (2001). Intravenous self-administration of the cannabinoid CB1 receptor agonist WIN 55,212-2 in rats. Psychopharmacology (Berl). 156, 410–416. 10.1007/s002130100734 11498718

[B65] Fontanilla D. Johannessen M. Hajipour A. R. Cozzi N. V. Jackson B. Ruoho A. E. (2010). NIH Public Access. Science, Vol. 323, 934–937. 10.1126/science.1166127 PMC2947205 19213917

[B66] Fraser F. (2014). New Psychoactive Substances – Evidence Review, Safer Communities Analytical Unit. Edinburgh: Scottish Government Social Research.

[B67] Gatch M. B. Forster M. J. (2014). Δ9-Tetrahydrocannabinol-like discriminative stimulus effects of compounds commonly found in K2/Spice. Behav. Pharmacol. 25, 750–757. 10.1097/FBP.0000000000000093 25325289 PMC4216610

[B68] Gatch M. B. Forster M. J. (2015). Δ9-Tetrahydrocannabinol-like effects of novel synthetic cannabinoids found on the gray market. Behav. Pharmacol. 26, 460–468. 10.1097/FBP.0000000000000150 26061356 PMC4497846

[B69] Gatch M. B. Rutledge M. A. Carbonaro T. Forster M. J. (2009). Comparison of the discriminative stimulus effects of dimethyltryptamine with different classes of psychoactive compounds in rats. Psychopharmacology (Berl). 204, 715–724. 10.1007/s00213-009-1501-z 19288085 PMC2865430

[B70] Gatch M. B. Taylor C. M. Forster M. J. (2013). Locomotor stimulant and discriminative stimulus effects of “bath salt” cathinones. Behav. Pharmacol. 24, 437–447. 10.1097/FBP.0b013e328364166d 23839026 PMC4183201

[B71] German C. L. Fleckenstein A. E. Hanson G. R. (2014). Bath salts and synthetic cathinones: an emerging designer drug phenomenon. Life Sci. 97, 2–8. 10.1016/j.lfs.2013.07.023 23911668 PMC3909723

[B72] Glass M. Dragunow M. Faull R. L. (1997). Cannabinoid receptors in the human brain: a detailed anatomical and quantitative autoradiographic study in the fetal, neonatal and adult human brain. Neuroscience 77, 299–318. 10.1016/S0306-4522(96)00428-9 9472392

[B73] González D. Ventura M. Caudevilla F. Torrens M. Farre M. (2013). Consumption of new psychoactive substances in a Spanish sample of research chemical users. Hum. Psychopharmacol. 28, 332–340. 10.1002/hup.2323 23881881

[B74] Goodman J. Packard M. G. (2015). The influence of cannabinoids on learning and memory processes of the dorsal striatum. Neurobiol. Learn. Mem. 125, 1–14. 10.1016/j.nlm.2015.06.008 26092091

[B75] Gregg R. A. Baumann M. H. Partilla J. S. Bonano J. S. Vouga A. Tallarida C. S.. (2015). Stereochemistry of mephedrone neuropharmacology: enantiomer-specific behavioural and neurochemical effects in rats. Br. J. Pharmacol. 172, 883–894. 10.1111/bph.12951 25255824 PMC4301696

[B76] Grim T. W. Wiebelhaus J. M. Morales A. J. Negus S. S. Lichtman A. H. (2015). Effects of acute and repeated dosing of the synthetic cannabinoid CP55,940 on intracranial self-stimulation in mice. Drug Alcohol Depend. 150, 31–37. 10.1016/j.drugalcdep.2015.01.022 25772438 PMC4601922

[B77] Gunderson E. W. Haughey H. M. Ait-Daoud N. Joshi A. S. Hart C. L. (2012). “Spice” and “K2” Herbal Highs: a case series and systematic review of the clinical effects and biopsychosocial implications of synthetic cannabinoid use in humans. Am. J. Addict. 21, 320–326. 10.1111/j.1521-0391.2012.00240.x 22691010

[B78] Hadlock G. C. Webb K. M. McFadden L. M. Chu P. W. Ellis J. D. Allen S. C.. (2011). 4-Methylmethcathinone (mephedrone): neuropharmacological effects of a designer stimulant of abuse. J. Pharmacol. Exp. Ther. 339, 530–536. 10.1124/jpet.111.184119 21810934 PMC3200001

[B79] Heimer L. Zahm D. S. Churchill L. Kalivas P. W. Wohltmann C. (1991). Specificity in the projection patterns of accumbal core and shell in the rat. Neuroscience 41, 89–125. 10.1016/0306-4522(91)90202-Y 2057066

[B80] Helander A. Beck O. Hägerkvist R. Hultén P. (2013). Identification of novel psychoactive drug use in Sweden based on laboratory analysis–initial experiences from the STRIDA project. Scand. J. Clin. Lab Invest. 73, 400–406. 10.3109/00365513.2013.793817 23692208

[B81] Helander A. Bäckberg M. Hultén P. Al-Saffar Y. Beck O. (2014). Detection of new psychoactive substance use among emergency room patients: results from the Swedish STRIDA project. Forensic Sci. Int. 243, 23–29. 10.1016/j.forsciint.2014.02.022 24726531

[B82] Herkenham M. Lynn A. B. Johnson M. R. Melvin L. S. de Costa B. R. Rice K. C. (1991). Characterization and localization of cannabinoid receptors in rat brain: a quantitative *in vitro* autoradiographic study. J. Neurosci. 11, 563–583. 1992016 10.1523/JNEUROSCI.11-02-00563.1991 PMC6575215

[B83] Hermanns-Clausen M. Kneisel S. Szabo B. Auwärter V. (2013). Acute toxicity due to the confirmed consumption of synthetic cannabinoids: clinical and laboratory findings. Addiction 108, 534–544. 10.1111/j.1360-0443.2012.04078.x 22971158

[B84] Hilário M. R. Clouse E. Yin H. H. Costa R. M. (2007). Endocannabinoid signaling is critical for habit formation. Front. Integr. Neurosci. 1:6. 10.3389/neuro.07.006.2007 18958234 PMC2526012

[B85] Hohmann N. Mikus G. Czock D. (2014). Effects and risks associated with novel psychoactive substances: mislabeling and sale as bath salts, spice, and research chemicals. Dtsch. Arztebl. Int. 111, 139–147. 10.3238/arztebl.2014.0139 24661585 PMC3965957

[B86] Hondebrink L. Nugteren-van Lonkhuyzen J. J. Van Der Gouwe D. Brunt T. M. (2015). Monitoring new psychoactive substances (NPS) in The Netherlands: data from the drug market and the Poisons Information Centre. Drug Alcohol Depend. 147, 109–115. 10.1016/j.drugalcdep.2014.11.033 25541244

[B87] Hwang J.-Y. Kim J.-S. Oh J.-H. Hong S.-I. Ma S.-X. Jung Y.-H.. (2015). The new stimulant designer compound pentedrone exhibits rewarding properties and affects dopaminergic activity. Addict. Biol. 10.1111/adb.12299 [Epub ahead of print]. 26290055

[B88] IACP (International Association of Chiefs of Police) (2012). The 2012 Annual Report of the Drug Recognition Expert Section, Alexandria, VA.

[B89] Iversen L. Gibbons S. Treble R. Setola V. Huang X.-P. Roth B. L. (2013). Neurochemical profiles of some novel psychoactive substances. Eur. J. Pharmacol. 700, 147–151. 10.1016/j.ejphar.2012.12.006 23261499 PMC3582025

[B90] Iversen L. White M. Treble R. (2014). Designer psychostimulants: pharmacology and differences. Neuropharmacology 87, 59–65. 10.1016/j.neuropharm.2014.01.015 24456744

[B91] Johnston L. D. O'Malley P. M. Bachman J. G. Schulenberg J. E. (2013). Monitoring the Future National Results on Adolescent Drug Use: Overview of Key Findings. Bethesda, MD: National Institute on Drug Abuse, 2013. Available online at www.monitoringthefuture.org

[B92] Justinova Z. Tanda G. Munzar P. Goldberg S. R. (2004). The opioid antagonist naltrexone reduces the reinforcing effects of Delta 9 tetrahydrocannabinol (THC) in squirrel monkeys. Psychopharmacology (Berl). 173, 186–194. 10.1007/s00213-003-1693-6 14668977

[B93] Karila L. Megarbane B. Cottencin O. Lejoyeux M. (2015). Synthetic cathinones: a new public health problem. Curr. Neuropharmacol. 13, 12–20. 10.2174/1570159X13666141210224137 26074740 PMC4462036

[B94] Karlsson L. Andersson M. Kronstrand R. Kugelberg F. C. (2014). Mephedrone, Methylone and 3,4-Methylenedioxypyrovalerone (MDPV) induce conditioned place preference in mice. Basic Clin. Pharmacol. Toxicol. 115, 411–416. 10.1111/bcpt.12253 24739011

[B95] Kehr J. Ichinose F. Yoshitake S. Goiny M. Sievertsson T. Nyberg F.. (2011). Mephedrone, compared with MDMA (ecstasy) and amphetamine, rapidly increases both dopamine and 5-HT levels in nucleus accumbens of awake rats. Br. J. Pharmacol. 164, 1949–1958. 10.1111/j.1476-5381.2011.01499.x 21615721 PMC3246659

[B96] Kelly J. P. (2011). Cathinone derivatives: A review of their chemistry, pharmacology and toxicology. Drug Test. Anal. 3, 439–453. 10.1002/dta.313 21755607

[B97] Kersten B. P. McLaughlin M. E. (2015). Toxicology and management of novel psychoactive drugs. J. Pharm. Pract. 28, 50–65. 10.1177/0897190014544814 25261428

[B98] Khullar V. Jain A. Sattari M. (2014). Emergence of New Classes of Recreational Drugs—Synthetic Cannabinoids and Cathinones. J. Gen. Intern. Med. 29, 1200–1204. 10.1007/s11606-014-2802-4 24553958 PMC4099455

[B99] Kikura-Hanajiri R. Kawamura N. U. M. Goda Y. (2014). Changes in the prevalence of new psychoactive substances before and after the introduction of the generic scheduling of synthetic cannabinoids in Japan. Drug Test. Anal. 6, 832–839. 10.1002/dta.1584 24573957

[B100] Koob G. F. Volkow N. D. (2010). Neurocircuitry of addiction. Neuropsychopharmacology 35, 217–238. 10.1038/npp.2009.110 19710631 PMC2805560

[B101] Kronstrand R. Brinkhagen L. Birath-Karlsson C. Roman M. Josefsson M. (2014). LC-QTOF-MS as a superior strategy to immunoassay for the comprehensive analysis of synthetic cannabinoids in urine. Anal. Bioanal. Chem. 406, 3599–3609. 10.1007/s00216-013-7574-x 24424965

[B102] Kwilasz A. J. Negus S. S. (2012). Dissociable effects of the cannabinoid receptor agonists Δ9-tetrahydrocannabinol and CP55940 on pain-stimulated versus pain-depressed behavior in rats. J. Pharmacol. Exp. Ther. 343, 389–400. 10.1124/jpet.112.197780 22892341 PMC3477211

[B103] Kyriakou C. Marinelli E. Frati P. Santurro A. Afxentiou M. Zaami S.. (2015). NBOMe : new potent hallucinogens – pharmacology, analytical methods, toxicities, fatalities : a review. Eur. Rev. Med. Pharmacol. Sci. 19, 3270–3281. 26400534

[B104] Le Roux G. Bruneau C. Lelièvre B. Deguigne M. B. Turcant A. Harry P.. (2015). Recreational phenethylamine poisonings reported to a French poison control center. Drug Alcohol Depend. 154, 46–53. 10.1016/j.drugalcdep.2015.05.048 26205314

[B105] Lecca D. Cacciapaglia F. Valentini V. Di Chiara G. (2006). Monitoring extracellular dopamine in the rat nucleus accumbens shell and core during acquisition and maintenance of intravenous WIN 55,212-2 self-administration. Psychopharmacology (Berl). 188, 63–74. 10.1007/s00213-006-0475-3 16850116

[B106] Liechti M. (2015). Novel psychoactive substances (designer drugs): overview and pharmacology of modulators of monoamine signaling. Swiss Med. Wkly. 145:w14043. 10.4414/smw.2015.14043 25588018

[B107] Lisek R. Xu W. Yuvasheva E. Chiu Y.-T. Reitz A. B. Liu-Chen L.-Y.. (2012). Mephedrone (“bath salt”) elicits conditioned place preference and dopamine-sensitive motor activation. Drug Alcohol Depend. 126, 257–262. 10.1016/j.drugalcdep.2012.04.021 22652295 PMC3478431

[B108] Loi B. Corkery J. M. Claridge H. Goodair C. Chiappini S. Gimeno Clemente C.. (2015). Deaths of individuals aged 16-24 years in the UK after usingmephedrone. Hum. Psychopharmacol. 30, 225–232. 10.1002/hup.2423 26216555

[B109] Lessin A. W. Long R. F. Parkes M. W. (1965). Central Stimulant Actions of Alpha-Alkyl Substituted Tryptamines in Mice. Br. J. Pharmacol. Chemother. 24, 49–67. 10.1111/j.1476-5381.1965.tb02079.x 14301999 PMC1704069

[B110] López-Arnau R. Martínez-Clemente J. Pubill D. Escubedo E. Camarasa J. (2012). Comparative neuropharmacology of three psychostimulant cathinone derivatives: butylone, mephedrone and methylone. Br. J. Pharmacol. 167, 407–420. 10.1111/j.1476-5381.2012.01998.x 22509960 PMC3481047

[B111] Lupica C. R. Riegel A. C. (2005). Endocannabinoid release from midbrain dopamine neurons: a potential substrate for cannabinoid receptor antagonist treatment of addiction. Neuropharmacology 48, 1105–1116. 10.1016/j.neuropharm.2005.03.016 15878779

[B112] Maas A. Wippich C. Madea B. Hess C. (2015). Driving under the influence of synthetic phenethylamines: a case series. Int. J. Legal Med. 129, 997–1003. 10.1007/s00414-015-1150-1 25618172

[B113] Macfarlane V. Christie G. (2015). Synthetic cannabinoid withdrawal: a new demand on detoxification services. Drug Alcohol Rev. 34, 147–153. 10.1111/dar.12225 25588420

[B114] Marshell R. Kearney-Ramos T. Brents L. K. Hyatt W. S. Tai S. Prather P. L.. (2014). *In vivo* effects of synthetic cannabinoids JWH-018 and JWH-073 and phytocannabinoid Δ(9)-THC in mice: inhalation versus intraperitoneal injection. Pharmacol. Biochem. Behav. 124, 40–47. 10.1016/j.pbb.2014.05.010 24857780 PMC4340656

[B115] Martellotta M. C. Cossu G. Fattore L. Gessa G. L. Fratta W. (1998). Self-administration of the cannabinoid receptor agonist WIN 55,212-2 in drug-naive mice. Neuroscience 85, 327–330. 10.1016/S0306-4522(98)00052-9 9622233

[B116] Martinotti G. Lupi M. Carlucci L. Cinosi E. Santacroce R. Acciavatti T.. (2015). Novel psychoactive substances: use and knowledge among adolescents and young adults in urban and rural areas. Hum Psychopharmacol. 30, 295–301. 10.1002/hup.2486 26216566

[B117] Marusich J. A. Grant K. R. Blough B. E. Wiley J. L. (2012). Effects of synthetic cathinones contained in “bath salts” on motor behavior and a functional observational battery in mice. Neurotoxicology 33, 1305–1313. 10.1016/j.neuro.2012.08.003 22922498 PMC3475178

[B118] Mátyás F. Urbán G. M. Watanabe M. Mackie K. Zimmer A. Freund T. F.. (2008). Identification of the sites of 2-arachidonoylglycerol synthesis and action imply retrograde endocannabinoid signaling at both GABAergic and glutamatergic synapses in the ventral tegmental area. Neuropharmacology 54, 95–107. 10.1016/j.neuropharm.2007.05.028 17655884 PMC2238033

[B119] Mavrikaki M. Markaki E. Nomikos G. G. Panagis G. (2010). Chronic WIN55,212-2 elicits sustained and conditioned increases in intracranial self-stimulation thresholds in the rat. Behav. Brain Res. 209, 114–118. 10.1016/j.bbr.2010.01.024 20097234

[B120] Maxwell J. C. (2014). Psychoactive substances–some new, some old: a scan of the situation in the U.S. Drug Alcohol Depend. 134, 71–77. 10.1016/j.drugalcdep.2013.09.011 24140401

[B121] Melis M. Sagheddu C. De Felice M. Casti A. Madeddu C. Spiga S.. (2014). Enhanced endocannabinoid-mediated modulation of rostromedial tegmental nucleus drive onto dopamine neurons in sardinian alcohol-preferring rats. J. Neurosci. 34, 12716–12724. 10.1523/JNEUROSCI.1844-14.2014 25232109 PMC4166158

[B122] Meririnne E. Kajos M. Kankaanpää A. Seppälä T. (2006). Rewarding properties of 1-benzylpiperazine, a new drug of abuse, in rats. Basic Clin. Pharmacol. Toxicol. 98, 346–350. 10.1111/j.1742-7843.2006.pto_243.x 16623856

[B123] Mills B. Yepes A. Nugent K. (2015). Synthetic Cannabinoids. Am. J. Med. Sci. 350, 59–62. 10.1097/MAJ.0000000000000466 26132518

[B124] Monteiro M. S. Bastos M. D. L. Guedes de Pinho P. Carvalho M. (2013). Update on 1-benzylpiperazine (BZP) party pills. Arch. Toxicol. 87, 929–947. 10.1007/s00204-013-1057-x 23685794

[B125] Motbey C. P. Clemens K. J. Apetz N. Winstock A. R. Ramsey J. Li K. M.. (2013). High levels of intravenous mephedrone (4-methylmethcathinone) self-administration in rats: neural consequences and comparison with methamphetamine. J. Psychopharmacol. 27, 823–836. 10.1177/0269881113490325 23739178

[B126] National Drug Intelligence Center (NDIC) (2011). National Drug Treat Assessment.

[B127] Negus S. S. Miller L. L. (2014). Intracranial self-stimulation to evaluate abuse potential of drugs. Pharmacol. Rev. 66, 869–917. 10.1124/pr.112.007419 24973197 PMC4081730

[B128] Nelson M. E. Bryant S. M. Aks S. E. (2014). Emerging drugs of abuse. Dis. Mon. 60, 110–132. 10.1016/j.disamonth.2014.01.001 24629403

[B129] Nichols D. E. (2004). Hallucinogens. Pharmacol. Ther. 101, 131–181. 10.1016/j.pharmthera.2003.11.002 14761703

[B130] NIDA (National Institute on Drug Abuse) (2012). Monitoring the Future 2012 Survey Results. Available online at: https://www.drugabuse.gov/related-topics/trends-statistics/infographics/monitoring-future-2012-survey-results (Accessed October 29, 2015).

[B131] Nishimura M. Sato K. (1999). Ketamine stereoselectively inhibits rat dopamine transporter. Neurosci. Lett. 274, 131–134. 10.1016/S0304-3940(99)00688-6 10553955

[B132] Ossato A. Canazza I. Trapella C. Vincenzi F. De Luca M. A. Rimondo C.. (2016). Effect of JWH-250, JWH-073 and their interaction on “tetrad,” sensorimotor, neurological and neurochemical responses in mice. Prog Neuropsychopharmacol. Biol Psychiatry. 15, 31–50. 10.1016/j.pnpbp.2016.01.007 26780169

[B133] Paillet-Loilier M. Cesbron A. Le Boisselier R. Bourgine J. Debruyne D. (2014). Emerging drugs of abuse: current perspectives on substituted cathinones. Subst. Abuse Rehabil. 5, 37–52. 10.2147/SAR.S37257 24966713 PMC4043811

[B134] Palamar J. J. Martins S. S. Su M. K. Ompad D. C. (2015). Self-reported use of novel psychoactive substances in a US nationally representative survey: Prevalence, correlates, and a call for new survey methods to prevent underreporting. Drug Alcohol Depend. 156, 112–119. 10.1016/j.drugalcdep.2015.08.028 26377051 PMC4633323

[B135] Panagis G. Mackey B. Vlachou S. (2014). Cannabinoid regulation of brain reward processing with an emphasis on the role of CB1 receptors: a step Back into the Future. Front. Psychiatry 5:92. 10.3389/fpsyt.2014.00092 25132823 PMC4117180

[B136] Papanti D. Schifano F. Botteon G. Bertossi F. Mannix J. Vidoni D.. (2013). “Spiceophrenia”: a systematic overview of “spice”-related psychopathological issues and a case report. Hum. Psychopharmacol. 28, 379–389. 10.1002/hup.2312 23881886

[B137] Paus T. (2005). Mapping brain maturation and cognitive development during adolescence. TrendsCogn Sci. 9, 60–68. 10.1016/j.tics.2004.12.008 15668098

[B138] Prosser J. M. Nelson L. S. (2012). The toxicology of bath salts: a review of synthetic cathinones. J. Med. Toxicol. 8, 33–42. 10.1007/s13181-011-0193-z 22108839 PMC3550219

[B139] Sanders B. Lankenau S. E. Bloom J. J. Hathazi D. (2008). “Research chemicals”: tryptamine and phenethylamine use among high-risk youth. Subst. Use Misuse. 43, 389–402. 10.1080/00952990701202970 18365939 PMC2536767

[B140] Santacroce R. Corazza O. Martinotti G. Bersani F. S. Valeriani G. Di Giannantonio M. (2015). Psyclones: a roller coaster of life? Hidden synthetic cannabinoids and stimulants in apparently harmless products. Hum. Psychopharmacol. 30, 265–271. 10.1002/hup.2410 26216561

[B141] Sañudo-Peña M. C. Tsou K. Delay E. R. Hohman A. G. Force M. Walker J. M. (1997). Endogenous cannabinoids as an aversive or counter-rewarding system in the rat. *Neurosci*. Lett. 223, 125–128. 9089689 10.1016/s0304-3940(97)13424-3

[B142] Schifano F. (2013). Novel psychoactive substances also known as ‘legal highs', in Annual Report of the Chief MedicalOfficer. Public Mental Health Priorities: Investing in the Evidence, ed Davies S. C (London: Department of Health), 259.

[B143] Schifano F. Corkery J. M. Cuffolo G. (2007). Smokable (“ice,” “crystal meth”) and non smokable amphetamine-type stimulants: Clinical pharmacological and epidemiological issues, with special reference to the UK. Ann. Ist Super. Sanita 43, 110–115. 17536161

[B144] Schifano F. Corkery J. Naidoo V. Oyefeso A. Ghodse H. (2010). Overview ofamphetamine-type stimulant mortality data–UK, 1997-2007. Neuropsychobiology 61, 122–130. 10.1159/000279302 20110737

[B145] Schifano F. Corkery J. Ghodse A. H. (2012). Suspected and confirmed fatalities associated with mephedrone (4-methylmethcathinone;‘meow meow') in the UK. J. Clin. Psychopharmacol. 32, 7104 10.1097/JCP.0b013e318266c70c 22926609

[B146] Schifano F. Orsolini L. Duccio Papanti G. Corkery J. M. (2015). Novel psychoactive substances of interest for psychiatry. World Psychiatry 14, 15–26. 10.1002/wps.20174 25655145 PMC4329884

[B147] Schindler C. W. Thorndike E. B. Goldberg S. R. Lehner K. R. Cozzi N. V. Brandt S. D.. (2015). Reinforcing and neurochemical effects of the “bath salts” constituents 3,4-methylenedioxypyrovalerone (MDPV) and 3,4-methylenedioxy-N-methylcathinone (methylone) in male rats. Psychopharmacology (Berl). 10.1007/s00213-015-4057-0 [Epub ahead of print]. 26319160 PMC4772144

[B148] Seely K. A. Prather P. L. James L. P. Moran J. H. (2011). Marijuana-based drugs: innovative therapeutics or designer drugs of abuse? Mol. Interv. 11, 36–51. 10.1124/mi.11.1.6 21441120 PMC3139381

[B149] Seely K. A. Brents L. K. Radominska-Pandya A. Endres G. W. Keyes G. S. Moran J. H. (2012). A major glucuronidated metabolite of JWH-018 is a neutral antagonist at CB1 receptors. Chem. Res. Toxicol. 39, 234–243. 10.1021/tx3000472 PMC3921679 22404317

[B150] Sidhpura N. Parsons L. H. (2011). Endocannabinoid-mediated synaptic plasticity and addiction-related behavior. Neuropharmacology 61, 1070–1087. 10.1016/j.neuropharm.2011.05.034 21669214 PMC3176941

[B151] Simmler L. D. Buser T. A. Donzelli M. Schramm Y. Dieu L.-H. Huwyler J.. (2013). Pharmacological characterization of designer cathinones *in vitro*. Br. J. Pharmacol. 168, 458–470. 10.1111/j.1476-5381.2012.02145.x 22897747 PMC3572571

[B152] Simmler L. D. Rickli A. Schramm Y. Hoener M. C. Liechti M. E. (2014). Pharmacological profiles of aminoindanes, piperazines, and pipradrol derivatives. Biochem. Pharmacol. 88, 237–244. 10.1016/j.bcp.2014.01.024 24486525

[B153] Simonato P. Corazza O. Santonastaso P. Corkery J. Deluca P. Davey Z.. (2013). Novel psychoactive substances as a novel challenge for health professionals: results from an Italian survey. Hum. Psychopharmacol. 28, 324–331. 10.1002/hup.2300 23881880

[B154] Smith J. P. Sutcliffe O. B. Banks C. E. (2015). An overview of recent developments in the analytical detection of new psychoactive substances (NPSs). Analyst 140, 4932–4948. 10.1039/C5AN00797F 26031385

[B155] Sogawa C. Sogawa N. Tagawa J. Fujino A. Ohyama K. Asanuma M.. (2007). 5-Methoxy-N,N-diisopropyltryptamine (Foxy), a selective and high affinity inhibitor of serotonin transporter. Toxicol. Lett. 170, 75–82. 10.1016/j.toxlet.2007.02.007 17382495

[B156] Solinas M. Panlilio L. V. Justinova Z. Yasar S. Goldberg S. R. (2006). Using drug-discrimination techniques to study the abuse-related effects of psychoactive drugs in rats. Nat Protoc. 1, 1194–1206. 10.1038/nprot.2006.167 17406402

[B157] Spaderna M. Addy P. H. D'souza D. C. (2013). Spicing things up: synthetic cannabinoids. Psychopharmacology (Berl) 228, 525–540. 10.1007/s00213-013-3188-4 23836028 PMC3799955

[B158] Sussman S. Skara S. Ames S. L. (2008). Substance abuse among adolescents. Substance Use Misuse 43, 1802–1828. 10.1080/10826080802297302 19016166

[B159] Tanda G. Pontieri F. E. Di Chiara G. (1997). Cannabinoid and heroin activation of mesolimbic dopamine transmission by a common mu1 opioid receptor mechanism. Science 276, 2048–2050. 10.1126/science.276.5321.2048 9197269

[B160] Tanda G. Munzar P. Goldberg S. R. (2000). Self-administration behavior is maintained by the psychoactive ingredient of marijuana in squirrel monkeys. Nat. Neurosci. 3, 1073–1074. 10.1038/80577 11036260

[B161] Teixeira-Gomes A. Costa V. M. Feio-Azevedo R. de Lourdes Bastos M. Carvalho F. Capela J. P. S. (2014). The neurotoxicity of amphetamines during the adolescent period. Int. J. Dev. Neurosci. 41, 1–18. 10.1016/j.ijdevneu.2014.12.001 25482046

[B162] Thomas S. Bliss S. Malik M. (2012). Suicidal ideation and self-harm following K2 use. J. Okla. State Med. Assoc. 105, 430–433. 23304900

[B163] Tittarelli R. Mannocchi G. Pantano F. Romolo F. S. (2015). Recreational use, analysis and toxicity of tryptamines. Curr. Neuropharmacol. 13, 26–46. 10.2174/1570159X13666141210222409 26074742 PMC4462041

[B164] Vigolo A. Ossato A. Trapella C. Vincenzi F. Rimondo C. Seri C.. (2015). Novel halogenated derivates of JWH-018: behavioral and binding studies in mice. Neuropharmacology 95, 68–82. 10.1016/j.neuropharm.2015.02.008 25769232

[B165] UNODC (2013). Global Smart Update 2013. Vienna.

[B166] UNODC (2014a). Early Warning Advisory on NPS. Vienna.

[B167] UNODC (2014b). World Drugs Report. Vienna.

[B168] UNODC (2015). The Challenge of Synthetic Drugs in East and South-East Asia and Oceania. Trends and Patterns of Amphetamine-type Stimulants and New Psychoactive Substances. World Drugs Report. Wien: Global SMART Programme.

[B169] Valente M. J. Guedes de Pinho P. de Lourdes Bastos M. Carvalho F. Carvalho M. (2014). Khat and synthetic cathinones: a review. Arch. Toxicol. 88, 15–45. 10.1007/s00204-013-1163-9 24317389

[B170] Valjent E. Maldonado R. (2000). A behavioural model to reveal place preference to delta 9-tetrahydrocannabinol in mice. Psychopharmacology 147, 436–438. 10.1007/s002130050013 10672638

[B171] Van Amsterdam J. Brunt T. van den Brink W. (2015). The adverse health effects of synthetic cannabinoids with emphasis on psychosis-like effects. J. Psychopharmacol. 29, 254–263. 10.1177/0269881114565142 25586398

[B172] Vlachou S. Nomikos G. G. Panagis G. (2005). CB1 cannabinoid receptor agonists increase intracranial self-stimulation thresholds in the rat. Psychopharmacology (Berl) 179, 498–508. 10.1007/s00213-004-2050-0 15821959

[B173] Vlachou S. Nomikos G. G. Stephens D. N. Panagis G. (2007). Lack of evidence for appetitive effects of Delta 9-tetrahydrocannabinol in the intracranial self-stimulation and conditioned place preference procedures in rodents. Behav. Pharmacol. 18, 311–319. 10.1097/FBP.0b013e3282186cf2 17551324

[B174] Volkow N. D. Fowler J. S. Wang G.-J. (2003). The addicted human brain: insights from imaging studies. J. Clin. Invest. 111, 1444–1451. 10.1172/JCI18533 12750391 PMC155054

[B175] Völlm B. A. de Araujo I. E. Cowen P. J. Rolls E. T. Kringelbach M. L. Smith K. A.. (2004). Methamphetamine activates reward circuitry in drug naïve human subjects. Neuropsychopharmacology 29, 1715–1722. 10.1038/sj.npp.1300481 15138439

[B176] Wang X. Dow-Edwards D. Keller E. Hurd Y. L. (2003). Preferential limbic expression of the cannabinoid receptor mRNA in the human fetal brain. Neuroscience 118, 681–694. 12710976 10.1016/s0306-4522(03)00020-4

[B177] Watterson L. R. Hood L. Sewalia K. Tomek S. E. Yahn S. Johnson C. T.. (2012). The reinforcing and rewarding effects of methylone, a synthetic cathinone commonly found in “Bath Salts.” J. Addict. Res. Ther. pii Suppl. 9:002. 10.4172/2155-6105.S9-002 24244886 PMC3828752

[B178] Watterson L. R. Kufahl P. R. Nemirovsky N. E. Sewalia K. Grabenauer M. Thomas B. F.. (2014). Potent rewarding and reinforcing effects of the synthetic cathinone 3,4-methylenedioxypyrovalerone (MDPV). Addict. Biol. 19, 165–174. 10.1111/j.1369-1600.2012.00474.x 22784198 PMC3473160

[B179] Wells D. L. Ott C. A. (2011). The “new” marijuana. Ann. Pharmacother. 45, 414–417. 10.1345/aph.1P580 21325097

[B180] Welter-Luedeke J. Maurer H. H. (2015). New psychoactive substances. Ther. Drug Monit. 38, 4–11. 10.1097/FTD.0000000000000240 26327309

[B181] Whelpton R. (2007). Speed, Ecstasy, Ritalin: the science of amphetamines. Br. J. Clin. Pharmacol. 63, 763–763. 10.1111/j.1365-2125.2006.02818.x

[B182] Wikström M. Holmgren P. Ahlner J. (2004). A2 (N-benzylpiperazine) a new drug of abuse in Sweden. J. Anal. Toxicol. 28, 67–70. 10.1093/jat/28.1.67 14987428

[B183] Wiley J. L. Marusich J. A. Martin B. R. Huffman J. W. (2012). 1-Pentyl-3-phenylacetylindoles and JWH-018 share *in vivo* cannabinoid profiles in mice. Drug Alcohol Depend. 123, 148–153. 10.1016/j.drugalcdep.2011.11.001 22127210 PMC3294131

[B184] Wiley J. L. Marusich J. A. Huffman J. W. (2014). Moving around the molecule: relationship between chemical structure and *in vivo* activity of synthetic cannabinoids. Life Sci. 97, 55–63. 10.1016/j.lfs.2013.09.011 24071522 PMC3944940

[B185] Winstock A. R. Barratt M. J. (2013). The 12-month prevalence and nature of adverse experiences resulting in emergency medical presentations associated with the use of synthetic cannabinoid products. Hum. Psychopharmacol. Clin. Exp. 28, 390–393. 10.1002/hup.2292 23881887

[B186] Winstock A. Schifano F. (2009). Disorders relating to the use of ecstasy, other ‘party drugs' and khat, in *New Oxford Textbook of Psychiatry* eds Gelder M. Andreasen N. Lopez-Ibor J. J. Geddes J. (Oxford: Oxford University Press), 494–502.

[B187] Winstock A. R. Mitcheson L. R. Deluca P. Davey Z. Corazza O. Schifano F. (2011). Mephedrone, new kid for the chop? Addiction 106, 154–161. 10.1111/j.1360-0443.2010.0330.x 20735367

[B188] Wood D. M. Heyerdahl F. Yates C. B. Dines A. M. Giraudon I. Hovda K. E. (2014). The european drug emergencies network (Euro-DEN). Clin. Toxicol. (Phila). 52, 239–241. 10.3109/15563650.2014.898771 24654801

[B189] Wood D. M. Sedefov R. Cunningham A. Dargan P. I. (2015). Prevalence of use and acute toxicity associated with the use of NBOMe drugs. Clin. Toxicol. 53, 85–92. 10.3109/15563650.2015.1004179 25658166

[B190] Wright M. J. Jr. Angrish D. Aarde S. M. Barlow D. J. Buczynski M. W. Creehan K. M.. (2012). Effect of ambient temperature on the thermoregulatory and locomotor stimulant effects of 4-methylmethcathinone in wistar and sprague-dawley rats. PLoS ONE 7:e44652. 10.1371/journal.pone.0044652 22952999 PMC3432134

[B191] Zahm D. S. Brog J. S. (1992). On the significance of subterritories in the “accumbens” part of the rat ventral striatum. Neuroscience 50, 751–767. 10.1016/0306-4522(92)90202-D 1448200

[B192] Zawilska J. B. (2015). “Legal Highs”–An Emerging Epidemic of Novel Psychoactive Substances. Int. Rev. Neurobiol. 120, 273–300. 10.1016/bs.irn.2015.02.009 26070762

[B193] Zimmermann U. S. Winkelmann P. R. Pilhatsch M. Nees J. A. Spanagel R. Schulz K. (2009). Withdrawal phenomena and dependence syndrome after the consumption of “spice gold.” Dtsch. Arztebl. Int. 106, 464–467. 10.3238/arztebl.2009.0464 19652769 PMC2719097

